# Transcriptomic Analysis Reveals Genes Associated with the Regulation of Peach Fruit Softening and Senescence during Storage

**DOI:** 10.3390/foods12081648

**Published:** 2023-04-14

**Authors:** Shaolei Guo, Ruijuan Ma, Jianlan Xu, Binbin Zhang, Mingliang Yu, Zhihong Gao

**Affiliations:** 1 Institute of Pomology, Jiangsu Academy of Agricultural Sciences/Jiangsu Key Laboratory for Horticultural Crop Genetic Improvement, Nanjing 210014, China; guoshaolei0305@126.com (S.G.); marj311@163.com (R.M.); jlxujaas1976@aliyun.com (J.X.); binbin1714@163.com (B.Z.); 2 College of Horticulture, Nanjing Agricultural University, Nanjing 210095, China

**Keywords:** auxin, peach, qRT-PCR, softening, transcriptomic analysis

## Abstract

Peach (*Prunus persica* (L.) Batsch) is a highly desirable fruit that is consumed around the world. However, the peach fruit is highly perishable after harvest, a characteristic that limits the distribution and supply to the market and causes heavy economic losses. Thus, peach fruit softening and senescence after harvest urgently need to be addressed. In the current study, transcriptomic analysis was performed to identify candidate genes associated with peach fruit softening and senescence, comparing peach fruit from cultivars with different flesh textures, namely melting and stony hard (SH) flesh textures during storage at room temperature. The mitogen-activated protein kinase signaling pathway-plant and plant hormone signal transduction pathways were associated with peach fruit softening and senescence according to the Venn diagram analysis and weighted gene co-expression network analysis. The expression levels of seven genes, including *Prupe.1G034300*, *Prupe.2G176900*, *Prupe.3G024700*, *Prupe.3G098100*, *Prupe.6G226100*, *Prupe.7G234800,* and *Prupe.7G247500*, were higher in melting peach fruit than in SH peach fruit during storage. Furthermore, the SH peach fruit softened rapidly after 1-naphthylacetic acid treatment, during which the levels of expression of these seven genes, determined by a quantitative reverse transcription polymerase chain reaction, were strongly induced and upregulated. Thus, these seven genes may play essential roles in regulating peach fruit softening and senescence.

## 1. Introduction

Peach (*Prunus persica* (L.) Batsch) is a crop of considerable economic value and has been widely cultivated around the world. Peach fruit is rich in phytonutrients, including many vitamins, amino acids, antioxidants, and proteins [1,2]. However, as a typical climacteric fruit, peach fruit is highly perishable during room temperature storage after harvest, and this feature limits its potential for long-distance transport to different markets and causes heavy economic losses, seriously restricting the development of the peach industry. Studying the physiological and molecular mechanisms behind peach fruit softening and senescence process will be important not only for developing fruit fresh-keeping techniques and achieving fruit supply at markets but also for the breeding of new peach varieties with storage tolerance.

Remodeling of the cell wall structure is regarded as the basis of changes in fruit firmness and texture [3] and is closely related to fruit softening and senescence. Numerous cell wall modifying enzymes, namely polygalacturonase [4,5], β-galactosidase [6,7,8], α-L-arabinofuranosidase [9], pectin lyase [10], and pectin methylesterase [11], are considered to play central roles in cell wall degradation, associated with fruit softening and senescence. Ethylene plays central roles in fruit softening and senescence [12,13,14]. Many transcription factors may regulate the activities of enzymes that catalyze the committed steps in ethylene biosynthesis to affect fruit softening and senescence, such as 1-aminocyclopropane-1-carboxylic acid synthase (ACS) and 1-aminocyclopropane-1-carboxylic acid oxidase (ACO). In apple fruit, the transcription factor MdMYC2 directly interacts with the promoter regions of *MdACS1* and *MdACO1* genes to upregulate their expression levels, which promotes ethylene production [15]. A new HD-ZIP transcription factor gene, *PpHB.G7*, was identified to bind to the promoter regions of *PpACS1* and *PpACO1* to regulate ACS and ACO activities and affect the ethylene production in peach fruit [16]. Significantly, various plant hormones, including abscisic acid (ABA) and auxin, can undergo crosstalk with ethylene biosynthesis in fruit ripening and softening [13,14,17]. In tomato, ABA induced the expression of ethylene synthesis genes, *SlACS* and *SlACO*, promoting fruit. However, the tomato fruit ripening and softening processes delayed treatment with fluridone or nordihydroguaiaretic acid, which block ABA accumulation. Thus, ABA may regulate the tomato fruit ripening and softening through ethylene [18]. The apple auxin response factor (ARF) MdARF5 can enhance the transcription levels of three ethylene synthesis genes, *MdACS3a*, *MdACS1,* and *MdACO1,* by directly binding to the promoter regions of these three genes, increasing the accumulation of ethylene production [19]. Interestingly, a peach YUCCA flavin mono-oxygenase (*PpYUC11*) gene, involved in the auxin biosynthesis pathway, was identified as being responsible for the stony hard (SH) peach trait by reducing indole-3-acetic acid (IAA) content in ripening fruit [20].

In addition, numerous transcription factors have been shown to be associated with fruit softening and senescence. In tomato, the *Nor* gene can regulate the protein abundance of polygalacturonase, pectate lyase, and cellulose synthase to affect fruit softening [21]. The two transcription factor genes, *AdEIL2* and *AdEIL3*, upregulated expression levels of *AdACO1* and *AdXET5* genes, affecting kiwifruit ripening and softening [22]. Additionally, an ethylene response factor *PpeERF2* in peach was identified to bind to the promoter region of a cell wall degradation gene, *PpePG1*, and regulate fruit ripening and softening by suppressing the expression of *PpePG1* [23]. Moreover, the FvWRKY48 transcription factor accelerates pectin degradation and fruit softening of wild strawberries by binding to the promoter of the pectate lyase gene *FvPLA* to upregulate its expression [24]. The characteristics of softening and senescence differ between peach fruit of different texture types. Generally, melting (M) fruit softens noticeably after harvest, whereas non-melting fruit exhibits slow softening after harvest and never melt, as the SH fruit is characterized by low-level ethylene production and is very firm during ripening (both on- and off-tree) [20,25]. Interestingly, after 1-naphthaleneacetic acid (NAA) treatment, the SH-type peach fruit produces a large amount of ethylene and softens rapidly [26]. No systematic transcriptomic analysis study of fruit softening and senescence with different peach flesh textures during room temperature storage has been conducted.

The peach fruit cultivar ‘HuJing Milu’(HJML) has been reported to soften rapidly, resulting from a large amount of ethylene production after harvest during room temperature storage, whereas ‘Xia Cui’(XC) and ‘Hua Yu’(HY) are SH-type cultivars with little ethylene production [6,20]. These findings indicate that SH peach will be a good model for investigating the effect of ethylene on fruit softening and senescence. In the current study, transcriptomic analysis was performed to identify candidate genes associated with peach fruit softening and senescence during room temperature storage using M peach and SH peaches. The identification of candidate genes would facilitate the selection of genes in breeding programs for reduced peach fruit softening and senescence and could provide a theoretical basis for the breeding of new peach varieties with a high tolerance of softening and achieve precise regulation of the postharvest quality maintenance of peach fruit.

## 2. Materials and Methods

### 2.1. Fruit Materials

Peaches (*Prunus persica* (L.) Batsch) from cultivars of ‘XC’ and ‘HJML’ from the orchard of the National Peach Germplasm Repository in Nanjing, Jiangsu, China, and ‘HY’ from an orchard in Beijing, China, were used in this study. Three healthy trees of each cultivar were managed in accordance with conventional cultivation measures. All peach fruit samples were randomly collected and checked to be of uniform commercial maturity with an undamaged fruit surface. An air-conditioned room with a temperature of 25 ± 1 °C and a relative humidity of 75–85% was used to store the fruit samples for six days in 2018. Samples were collected every three days during storage, with 15 fruits being sampled at each date for RNA-seq, and five fruits served as one biological replicate. In addition, ‘XC’ fruit samples at commercial maturity were treated with NAA, based on the method of Tatsuki et al. [26]. Fruits were sprayed with 1mM NAA (Sangon Biotech, Shanghai, China) containing 0.01% (*w*/*v*) Tween 20 (Sangon Biotech, Shanghai, China) every 24 h for seven days, while control fruits were sprayed with 0.01% (*w*/*v*) Tween 20 without NAA. All ‘XC’ fruits were stored in an air-conditioned room with a temperature of 25 ± 1 °C and a relative humidity of 75–85% for eight days in 2019. Samples were collected every two days, and 15 fruits were sampled at each date to determine ethylene production and fruit firmness, with five fruits serving as each biological replicate. All experiments were carried out at the Jiangsu Academy of Agricultural Sciences. After measurements were taken, the pulp flesh was rapidly frozen in liquid nitrogen and stored in an −80 °C refrigerator until needed for further analysis.

### 2.2. Ethylene Production and Fruit Firmness

Ethylene production measurement and fruit firmness determination were based on the methods of Guo et al. [27]. Briefly, ethylene production at each stage was measured by placing peach fruits in an airtight container for 2 h at 25 °C, and a syringe was used to obtain a 1 mL gas sample from the headspace of the airtight container. Immediately, the gas sample was analyzed by a gas chromatograph (Agilent 7890A, Santa Clara, CA, USA) with an HP-Plot q column (20 m × 0.53 mm × 20 µm). The injector and detector temperatures were 220 °C, and the column temperature was 40 °C. The TA. XT. Plus Texture Analyser (Stable Micro System, Godalming, UK) was used to measure fruit firmness. The expression unit of ethylene production was μL kg^−1^ h^−1^ and fruit firmness was kg/cm^2^.

### 2.3. RNA Sequencing Library Preparation

Total RNA was isolated from the peach fruit pulp sample using the RNAprep Pure Plant Kit (polysaccharide- and polyphenolic-rich) (TIANGEN, Beijing, China), according to the manufacturer’s instructions. Three independent biological replicates were used for RNA extraction. The total RNA quality and quantity were verified using a NanoDrop spectrophotometer and an Agilent 2100 Bioanalyzer (Thermo Fisher Scientific, Waltham, MA, USA), respectively. Briefly, the mRNA was fragmented into small pieces after purification by Oligo(dT)-attached magnetic beads. Reverse transcription was performed to obtain the first-strand cDNA using random hexamer primers, and a second-strand cDNA synthesis reaction was then undertaken. Subsequently, A-Tailing Mix and RNA Index Adapters were added to the cDNA. This was followed by polymerase chain reaction (PCR) amplification, and the Ampure XP Beads were used to purify the PCR products, and then the PCR products were eluted in an elution buffer solution. Afterward, the double-stranded PCR products underwent the reaction of heat-denatured and circularized to obtain the single-strand circle DNA as the final library. The final library was amplified using phi29 to achieve DNA nanoballs, which were loaded into the patterned nanoarray. Lastly, pair-end 100 base reads were obtained on the BGISEQ-500 platform (Beijing Genomic Institute, Shenzhen, China).

### 2.4. Data Filtering and Analysis

SOAPnuke (v1.4.0) [28] was used to obtain high-quality reads by filtering out the low-quality reads, reads containing the sequencing adapter, reads where the unknown base (‘N’ base) ratio was more than 5%, and reads with >20% nucleotides with base quality <5. Subsequently, the clean reads remaining after filtering were stored in FASTQ format. The clean reads were mapped to the reference genome Peach v2.0.a1 (v2.1) [29] using HISAT2 (v2.1.0) software [30]. Statistics of the clean reads are listed in Supplementary File S1. Differentially expressed genes (DEGs) were identified using DEGseq software [31], with a fold change ≥2 and a Q-value ≤ 0.001.

The Phyper, according to the hypergeometric test, was performed on the Kyoto Encyclopedia of Genes and Genomes (KEGG) pathway enrichment analysis of annotated DEGs [32]. And the annotated DEGs were analyzed by Gene Ontology (GO) classification [32]. Gene expression was presented as the fragments per kilobase of the exon model per million mapped fragments (FPKM) method. Screening conditions for upregulated genes were as follows: Log2 (HJML 3 d/HJML 0 d) ≥ 1, Log2 (HJML 3 d/XC 3 d) ≥ 1, Log2 (HJML 6 d/XC 6 d) ≥ 1, Log2 (HJML 3 d/HY 3 d) ≥ 1, Log2 (HJML 6 d/HY 6 d) ≥ 1, and HJML 6 d FPKM ≥ 5. Conditions for downregulated genes were as follows: Log2 (HJML 3 d/HJML 0 d) ≤ −1, Log2 (HJML 3 d/XC 3 d) ≤ −1, Log2 (HJML 6 d/XC 6 d) ≤ −1, Log2 (HJML 3 d/HY 3 d) ≤ −1, Log2 (HJML 6 d/HY 6 d) ≤ −1, XC 0 d FPKM ≥ 5, and HY 0 d FPKM ≥ 5. Additionally, weighted gene co-expression network analysis (WGCNA) was performed as previously described using WGCNA (v1.48, Gene Frac Threshold 0.5) [33].

### 2.5. The Expression Analysis of Candidate Genes

The PrimeScript™ RT reagent Kit with a gDNA Eraser (TaKaRa, Dalian, China) was used to synthesize cDNA from total RNA. To validate the peach transcriptome data, a quantitative reverse transcription polymerase chain reaction (qRT-PCR) was performed using TB Green^®^ Premix Ex Taq^TM^ II (Tli RNaseH Plus) (TaKaRa) on a 7500 Real-Time PCR System (Applied Biosystems, Carlsbad, CA, USA). The primers of qRT-PCR were designed using the NCBI/Primer-BLAST online server and listed in Supplementary File S2. Each PCR reaction was prepared in a 20 μL mixture volume containing diluted cDNA (1 μL), forward primer (0.4 μL), reverse primer (0.4 μL), TB green premix (2X, 10 μL), ROX reference dye II (0.4 μL), and RNase-free water (7.8 μL). The reaction was carried out under the following conditions: an initial denaturation step at 95 °C for 30 s, 40 cycles of 95 °C for 5 s, and 60 °C for 34 s. The relative expression level of each gene was normalized relative to the *translation elongation factor 2* (*TEF2*) gene [34] and detected by the 2^−ΔΔCt^ method [35].

### 2.6. Statistical Analysis

Microsoft Excel 2016 was used to calculate standard errors (SEs). Origin 9.1 was used to produce graphs. SPSS 19.0 (SPSS, Chicago, IL, USA) was used to determine the significant differences between experimental data.

## 3. Results

### 3.1. Identification of Candidate Genes by a Venn Diagram

**Figure 1:**
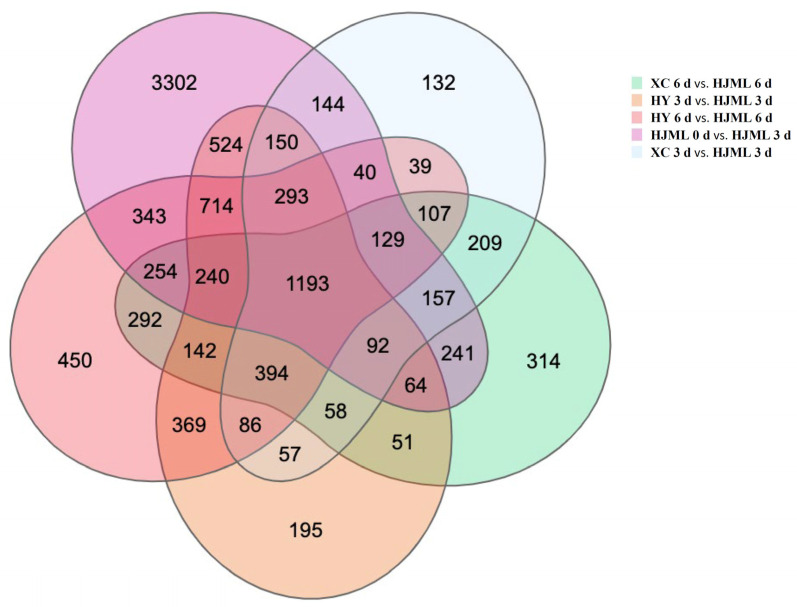
Venn diagram of differentially expressed genes between peach cultivars ‘HJML’, ‘HY’, and ‘XC’. ‘XC’ represents ‘Xia Cui’, ‘HY’ represents ‘Hua Yu’, and ‘HJML’ represents ‘Hujing Milu’.

Melting peach fruits have been reported to soften rapidly as a result of the release of a large amount of ethylene production after harvest during room temperature storage, whereas SH-type cultivars soften slowly with little ethylene production. Thus, Venn diagram analysis was performed at 3 d and 6 d during the storage period between one melting cultivar ‘HJML’ and two SH-type cultivars, ‘HY’ and ‘XC’. The melting cultivar ‘HJML’ was also analyzed from 0 d to 3 d to identify genes associated with peach fruit softening and senescence. The combinations ‘HJML’ 0 d vs. ‘HJML’ 3 d, ‘XC’ 3 d vs. ‘HJML’ 3 d, ‘XC’ 6 d vs. ‘HJML’ 6 d, ‘HY’ 3 d vs. ‘HJML’ 3 d, and ‘HY’ 6 d vs. ‘HJML’ 6 d were used for Venn diagram analysis (Figure 1). A total of 1193 putative DEGs were identified between the M peach ‘HJML’ and the SH peach cultivars ‘XC’ and ‘HY’ from a Venn analysis by group comparisons, which may be related to peach fruit softening and senescence. Subsequently, 159 upregulated genes and 365 downregulated genes were identified, which are listed in Supplementary File S3 and Supplementary File S4, according to the screening conditions.

### 3.2. Functional Analysis of Candidate Genes

**Figure 2:**
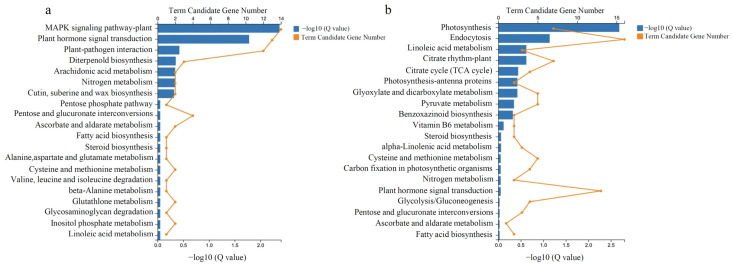
The Kyoto Encyclopedia of Genes and Genomes pathway enrichment of differentially expressed genes. (**a**) The KEGG pathway enrichment of DEGs from upregulated candidate genes. (**b**) The KEGG pathway enrichment of DEGs from downregulated candidate genes.

The upregulated genes were annotated in the GO database and were categorized into twenty-eight functional groups, including thirteen in biological processes, eight in cellular components, and seven in molecular functions (Supplementary File S5). Furthermore, the 159 upregulated genes were then annotated in the KEGG pathway database, and the top 20 most highly enriched pathways are shown in Figure 2a. Remarkably, 14 upregulated genes were found in the mitogen-activated protein kinase (MAPK) signaling pathway-plant (ko04016) and 13 genes were found in the plant hormone signal transduction pathway (ko04075); the other 18 pathways are listed in Figure 2a. The 365 downregulated genes were classified into twenty-six functional groups in the GO database, including eleven in biological processes, ten in cellular components, and five in molecular functions (Supplementary File S5). Photosynthesis was the most highly enriched of the pathways, and the top 20 most highly enriched pathways are shown in Figure 2b. The fold change and function annotation based on the reference genome of upregulated genes in the top five enriched pathways are shown in Table 1, while the corresponding downregulated genes are shown in Table 2. The 14 upregulated genes in the MAPK signaling pathway-plant (ko04016) and the 13 upregulated genes in the plant hormone signal transduction (ko04075) pathway were associated with ethylene biosynthesis and signal transduction, which may participate in fruit softening and senescence process. The BGI_novel sequence is listed in Supplementary File S6.

### 3.3. Weighted Gene Co-Expression Network Analysis of Candidate Genes

**Figure 3:**
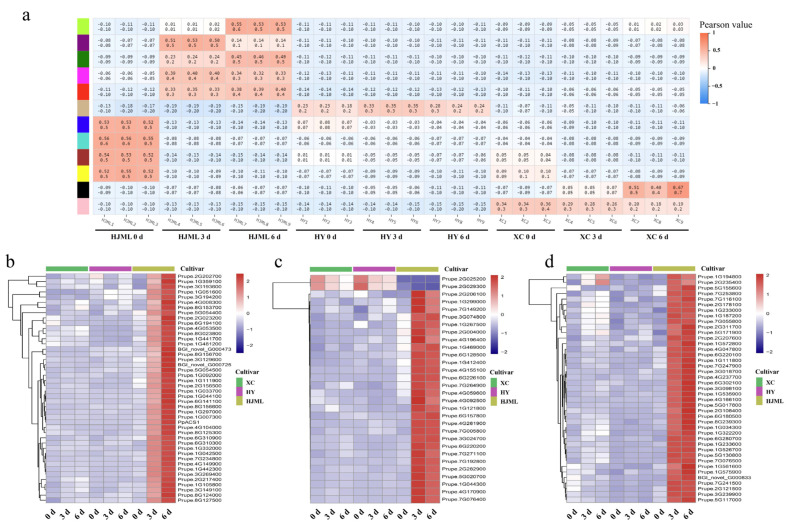
Weighted gene co-expression network analysis. (**a**) Correlation analysis between co-expression gene modules and phenotype. (**b**–**d**) Heatmaps of co-expression network analysis of stage-specific modules by RNA-Seq data: b represents the green module, c represents the magenta module, and d represents the red module. ‘XC’ represents ‘Xia Cui’, ‘HY’ represents ‘Hua Yu’, and ‘HJML’ represents ‘Hujing Milu’.

**Figure 4:**
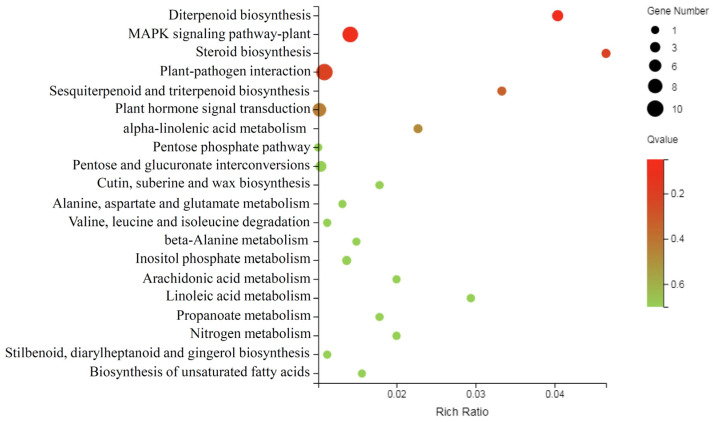
The Kyoto Encyclopedia of Genes and Genomes pathway enrichment of genes from the green, magenta, and red modules.

WGCNA was carried out to identify the hub genes regulating peach fruit softening and senescence. The M-type peach fruit released a larger amount of ethylene and softened noticeably after harvest, whereas the fruit from the two SH-type peach cultivars was characterized by their low ethylene production and very firm during ripening (both on- and off-tree) [20,25]. In Figure 3, the green, red, and magenta modules with the highest phenotypic correlations were selected for further analysis based on fruit ethylene production after harvest. A total of 115 genes were identified in three modules and were annotated in the KEGG database. The pathways of diterpenoid biosynthesis, the MAPK signaling pathway-plant, steroid biosynthesis, plant–pathogen interaction, sesquiterpenoid and triterpenoid biosynthesis, and plant hormone signal transduction were all highly enriched (Figure 4). Ethylene plays a central role in fruit softening and senescence, with the MAPK signaling pathway-plant also being associated with ethylene biosynthesis [36,37]. Nine genes in the MAPK signaling pathway-plant and seven genes in the plant hormone signal transduction pathways were identified for further study, while fourteen and thirteen genes were also identified by Venn diagram analysis in the MAPK signaling pathway-plant and plant hormone signal transduction pathway, respectively. We have reasons to believe that genes in the above two pathways may participate in the process of the regulation of fruit softening and senescence through ethylene biosynthesis and/or signal transduction. A total of 20 candidate genes were identified for further analysis.

### 3.4. FPKM Analysis of the Candidate Genes

**Figure 5:**
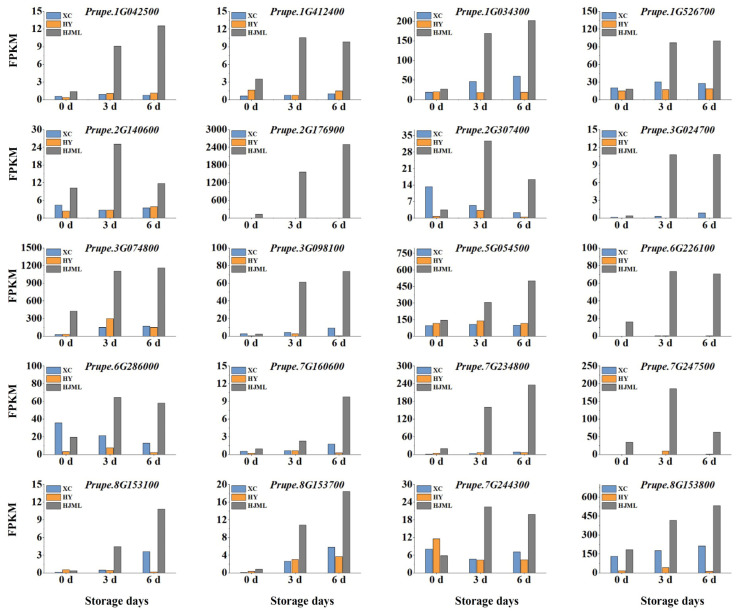
The expression analysis of candidate genes by RNA-seq technology. The *X*-axis represents the number of storage days after harvest whereas the *Y*-axis represents FPKM of genes. ‘XC’ represents ‘Xia Cui’, ‘HY’ represents ‘Hua Yu’, and ‘HJML’ represents ‘Hujing Milu’.

The gene expression levels of the 20 candidate genes were identified by Venn diagram analysis and the WGCNA method, and the gene expression level was analyzed by RNA-seq technology (Figure 5). The expression levels of the 20 candidate genes were higher during room temperature storage in the M-type peach ‘HJML’ than in SH peach ‘XC’ and ‘HY’. Significantly, the gene *PpACS1* (*Prupe.2G176900*) plays a crucial role in ethylene synthesis, and its suppressed expression was associated with a low level of ethylene production, resulting in the suppression of fruit softening in SH peach cultivars [38]. Interestingly, 12 genes showed similar expression patterns to *PpACS1* between the M-type peach ‘HJML’ and the SH-type peach cultivars (‘XC’ and ‘HY’), namely *Prupe.1G034300*, *Prupe.1G042500*, *Prupe.1G526700*, *Prupe.3G024700*, *Prupe.3G074800*, *Prupe.3G098100*, *Prupe.5G054500*, *Prupe.7G160600*, *Prupe.7G234800*, *Prupe.8G153100*, *Prupe.8G153700* and *Prupe.8G153800*. The expressions of *Prupe.1G412400*, *Prupe.2G140600*, *Prupe.2G307400*, *Prupe.6G226100*, *Prupe.6G286000*, *Prupe.7G244300,* and *Prupe.7G247500* increased at 3 d of postharvest storage in the M-type peach ‘HJML’ but declined by the end of storage (Figure 5). The FPKM and functional annotations of these 20 candidate genes related to softening and senescence are listed in Supplementary File S7.

### 3.5. The Effect of NAA Treatment on Candidate Gene Expression

**Figure 6:**
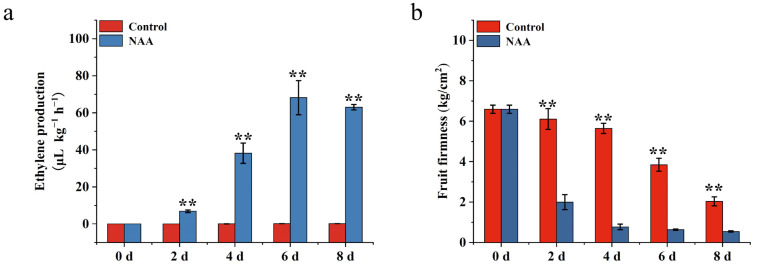
Ethylene production and fruit firmness analysis of ‘XC’ peach fruit after NAA treatment during room-temperature storage. (**a**) Ethylene production. (**b**) Fruit firmness. The *X*-axis represents the days of storage after harvest. Data are means ± SEs (*n* = 3), and significant differences (*p* < 0.01) by Student’s *t*-test between means are indicated by the symbol **.

**Figure 7:**
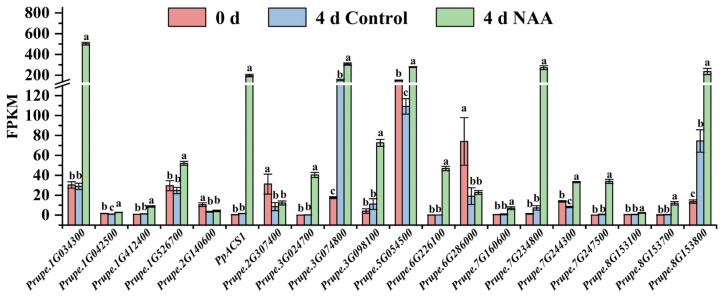
Gene expression analysis by transcriptome analysis of 20 candidate genes in ‘XC’ fruit treated with NAA. ‘XC’ represents ‘Xia Cui’; samples with different letters indicate significant differences at *p* < 0.05 using Duncan’s new multiple range test.

**Figure 8:**
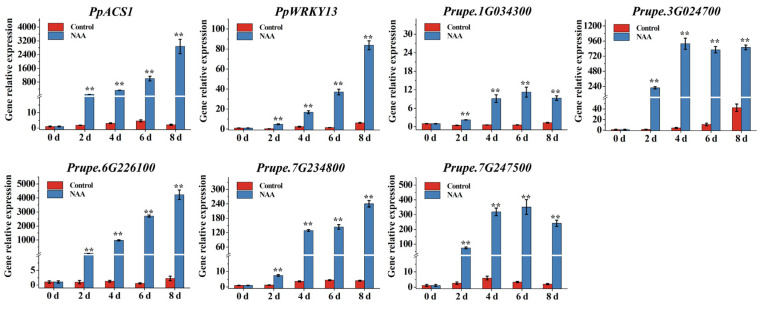
The relative expression analysis by qRT-PCR of candidate genes in ‘XC’ fruit treated with NAA. The *X*-axis represents the number of days after harvest, whereas the *Y*-axis represents the relative gene expression levels. Data are means ± SEs (*n* = 3). ‘XC’ represents ‘Xia Cui’; significant differences (*p* < 0.01) by Student’s *t*-test between means are indicated by the symbol **.

SH-type peach fruit can be induced to release a large amount of ethylene and achieve rapid softening in response to NAA treatment. In the present study, the SH-type peach cultivar ‘XC’ was used to analyze the candidate gene expression after NAA treatment. The control ‘XC’ fruit maintained fruit firmness (0–4 d), and hardly synthesized any ethylene during room temperature storage (Figure 6), whereas the firmness of fruit treated with NAA declined markedly after two days and retained a significantly lower firmness than that of the control fruit during storage. Ethylene production of ‘XC’ fruit was induced by NAA treatment after two days, with a pattern of increased production during storage (Figure 6). Generally, after NAA treatment, the SH-type cultivar ‘XC’ exhibited softening characteristics similar to those of M-type peach; thus, these materials would be extremely suitable for the identification of candidate genes associated with fruit softening and senescence. The expression levels of the 20 candidate genes identified earlier as being related to softening and senescence were analyzed by RNA-Seq using SH-type peach ‘XC’ with NAA treatment (FPKM data were listed in Supplementary File S8). Expression levels of seventeen of the twenty genes in ‘XC’ fruit during room temperature storage were altered in response to NAA treatment, whereas those of the remaining three genes (*Prupe.2G140600*, *Prupe.2G307400*, *Prupe.6G286000*) were not affected by NAA treatment (Figure 7). Of the seventeen genes, six genes, namely *Prupe.1G042500*, *Prupe.1G412400*, *Prupe.7G160600*, *Prupe.7G244300*, *Prupe.8G153100,* and *Prupe.8G153700*, exhibited lower expression levels, whereas four genes (*Prupe.1G526700*, *Prupe.3G074800*, *Prupe.5G054500,* and *Prupe.7G244300*) in ‘XC’ peach fruit were slightly upregulated by NAA treatment. On the other hand, the expression levels of seven genes (*Prupe.1G034300*, *PpACS1*, *Prupe.3G024700*, *Prupe.3G098100* (*PpWRKY13*), *Prupe.6G226100*, *Prupe.7G234800,* and *Prupe.7G247500*) were strongly induced and significantly upregulated in ‘XC’ peach fruit treated with NAA in comparison with control ‘XC’ fruit. Furthermore, qRT-PCR of these seven genes showed that they are strong candidates for genes positively associated with peach fruit softening and senescence (Figure 8).

## 4. Discussion

M-type peach fruit and SH-type peach are good models for investigating the effect of ethylene on fruit softening and senescence. Ethylene is a plant hormone with diverse functions, which involve various plant biology processes including germination, plant growth, organ senescence, and fruit ripening [39,40,41]. MAPK modules play essential roles in the transduction of developmental and environmental signals through the phosphorylation of other kinases, other enzymes, transcription factors, or other downstream signaling targets [42]. Studies have shown that MAPKs are also involved in ethylene biosynthesis and signaling [37,42,43]. In the present study, seven genes identified from the MAPK signaling pathway-plant and plant hormone signal transduction pathways may be associated with peach fruit softening and senescence, according to the Venn diagram and WGCNA analyses by the RNA-Seq technology, with the differential softening and senescence characteristics of peach fruit (M-type peach fruit and SH-type peach fruit) during room temperature storage.

Seven genes were identified from the MAPK signaling pathway-plant and plant hormone signal transduction pathways, in which expression levels were strongly induced and upregulated in ‘XC’ peach fruit after NAA treatment. *PpACS1* encodes the ACS, one of the enzymes which catalyze the synthesis of ethylene and is responsible for ethylene production by climacteric fruit [38,44]. Interestingly, the high expression level of *PpACS1* in M-type peach fruit results in a large amount of ethylene, followed by the rapid softening of the fruit [38]. The very low ethylene production in SH-type peach fruit may contribute to the maintenance of the fruit hardness resulting from the suppressed transcription of *PpACS1* [12,38,45,46]. Thus, the *PpACS1* was confirmed to play a central role in regulating peach fruit softening and senescence through the control of ethylene synthesis.

Additionally, the *Prupe.1G034300* gene was functionally annotated as ethylene response sensor 2-related (Supplementary File S7), and *Prupe.1G034300* showed expression patterns similar to *PpACS1* between the M-type peach ‘HJML’ and the SH-type peach (‘XC’ or ‘HY’). The increased expression level with fruit softening and senescence was consistent with a report examining pears and persimmons [47,48]. The high level of gene expression in M-type peaches may be due to the large amount of ethylene required for the ethylene receptor, which needs time to dissociate after binding with ethylene [49]. The low expression level in SH-type peach fruit, in association with low ethylene production, may result from insufficient ethylene being available with which to bind. Thus, the ethylene receptor gene *Prupe.1G034300* may participate in the process of peach fruit softening and senescence through ethylene signal transduction.

Three genes (*Prupe.6G226100*, *Prupe.7G234800,* and *Prupe.7G247500*) were functionally annotated as being associated with auxin response (Supplementary File S7). The gene *Prupe.6G226100* is functionally annotated as Gretchen Hagen 3 (GH3) and is associated with auxin homeostasis, whereas the other two genes (*Prupe.7G234800* and *Prupe.7G247500*) are functionally annotated as being associated with auxin/indole-3-acetic acid (Aux/IAA). The early or primary auxin-response genes consist of three major classes of genes, known as GH3, Aux/IAA, and the small auxin-up RNAs (SAURs) [50,51,52]. Generally, free auxin has biological activity, and it can be conjugated with sugars, amino acids, or peptides to generate auxin storage forms in plants [53]. The GH3 family gene members encode GH3 enzymes that catalyze the conjugation of amino acids to IAA, which may maintain the balance of free auxin to regulate the various life processes of plants [51,54]. Actually, the IAA concentration is higher in M-type peach fruit than in SH-type peach fruit, which is consistent with the system 2 ethylene production during the ripening period [26]. Additionally, the expression of *Prupe.6G226100* is higher in M-type peach than in SH-type peach fruit, suggesting that *Prupe.6G226100* may have an influence on regulating fruit softening and senescence by maintaining the balance of free and bound auxin. Degradation of Aux/IAA proteins by way of the ubiquitin/26S proteasome and the ARF activity is derepressed; thus, ARF proteins regulate the subsequent expression of auxin-responsive genes in the auxin-mediated transcription activation [55,56]. Overexpression of an Aux/IAA gene *PpIAA19* (*prupe.3G074800*) in tomato cultivar ‘Micro-Tom’ suggested that this gene was not related to the ripening of tomato fruit [57]. However, an Aux/IAA gene, *Prupe.7G234800* (*PpIAA1*), enhanced the transcription of the *PpACS1* gene and the cell wall degradation gene *PpPG1* in peach fruit, with overexpression of *PpIAA1* in tomatoes resulting in transgenic fruit softening more rapidly than the wild type [46]. Thus, the Aux/IAA gene *Prupe.7G247500* may have a similar function, and the function of this gene needs further analysis.

Three WRKY transcription factor genes (*Prupe.2G307400*, *PpWRKY13,* and *Prupe.6G286000*) were identified from the MAPK signaling pathway-plant and plant hormone signal transduction pathways. Significantly, the expression of *PpWRKY13* was strongly upregulated in ‘XC’ peach fruit in response to NAA treatment during the subsequent rapid softening process. WRKY transcription factors, as activators and inhibitors of transcription, are widely involved in the regulation of stress response, hormone signal transduction, and fruit ripening and senescence [24,58,59,60]. Significantly, WRKY transcription factors can bind to the promoter of key genes in the ethylene biosynthesis pathway to mediate ethylene biosynthesis. The gene *VvWRKY13* from *Vitis vinifera* was found to mediate the expression of genes *ACS2* and *ACS8* in ethylene biosynthesis [61]. Chromatin-immunoprecipitation assays revealed that WRKY33 regulated the expression of *ACS2* and *ACS6* by binding to the promoter regions of these genes in *Arabidopsis* [62]. Furthermore, WRKY transcription factors may affect fruit softening and senescence by regulating enzymes related to cell wall degradation. Based on one report, the wild strawberry WRKY transcription factor *FvWRKY48* binds to the promoter region of the corresponding pectate lyase gene *FvPLA* to control the pectin degradation and fruit softening in *Fragaria vesca* [24]. Thus, *PpWRKY13* may regulate the peach fruit softening and senescence by mediating the expression of key enzymes in ethylene biosynthesis and/or the enzymes related to cell wall degradation.

## 5. Conclusions

The expression of seven genes, including *Prupe.1G034300*, *Prupe.2G176900*, *Prupe.3G024700*, *Prupe.3G098100*, *Prupe.6G226100*, *Prupe.7G234800,* and *Prupe.7G247500,* were upregulated in M-type peach fruit relative to SH-type peach fruit and were strongly induced and upregulated in SH-type peach fruit after NAA treatment, which stimulated fruit flesh softening during room temperature storage. The transcriptomic analysis revealed that these seven genes are considered to be candidate genes associated with peach fruit softening and senescence.

## Figures and Tables

**Table 1:** Functional classification of upregulated genes.

Gene ID	Fold Change					Function Description
	log2(HJML 3 d/HJML 0 d)	log2(HJML 3 d/XC 3 d)	log2(HJML 6 d/XC 6 d)	log2(HJML 3 d/HY 3 d)	log2(HJML 6 d/HY 6 d)	
MAPK signaling pathway-plant						
*Prupe.1G034300*	2.65	1.86	1.75	3.19	3.40	Ethylene response sensor 2-related
*Prupe.1G042500*	2.72	3.30	4.07	3.05	3.48	Phosphate transporter pho1
*Prupe.1G412400*	1.58	3.81	3.32	3.84	2.86	Uncharacterized conserved protein
*Prupe.1G526700*	2.42	1.66	1.87	2.49	2.41	Ovarian cancer gene-2 protein-related//subfamily not named
*Prupe.2G176900*	3.63	8.62	8.55	8.29	11.01	1-aminocyclopropane-1-carboxylate synthase-like protein 1
*Prupe.2G307400*	3.23	2.62	2.88	3.33	5.42	WRKY DNA -binding domain (WRKY)
*Prupe.3G024700*	4.91	5.19	3.63	8.49	8.42	Receptor-like protein 3-related
*Prupe.3G098100*	4.69	3.80	3.00	4.42	8.17	WRKY transcription factor 40-related
*Prupe.5G054500*	1.07	1.51	2.34	1.13	2.10	Serine/threonine kinase//subfamily not named
*Prupe.6G286000*	1.73	1.61	2.19	3.10	4.76	WRKY transcription factor 33 (WRKY33)
*Prupe.7G244300*	1.95	2.26	1.49	2.36	2.15	EIN3-binding F-box protein (EBF1_2)
*Prupe.8G153100*	3.58	3.21	1.60	3.40	6.01	Pathogenesis-related protein 1 (PR1)
*Prupe.8G153700*	3.69	2.04	1.68	1.83	2.33	Pathogenesis-related protein 1 (PR1)
*Prupe.8G153800*	1.17	1.25	1.33	3.30	5.37	Pathogenesis-related protein 1 (PR1)
Plant hormone signal transduction						
*Prupe.1G034300*	2.65	1.86	1.75	3.19	3.40	Ethylene response sensor 2-related
*Prupe.1G042500*	2.72	3.30	4.07	3.05	3.48	Phosphate transporter pho1
*Prupe.2G140600*	1.28	3.25	1.80	3.24	1.63	Auxin-regulated protein-related
*Prupe.3G074800*	1.36	2.89	2.77	1.88	2.94	Auxin-responsive protein IAA19-related
*Prupe.5G054500*	1.07	1.51	2.34	1.13	2.10	Serine/threonine kinase//subfamily not named
*Prupe.6G226100*	2.17	8.87	11.13	9.03	9.16	Auxin-responsive GH3 gene family (GH3)
*Prupe.7G160600*	1.25	1.93	2.49	1.86	5.26	Camp-response element binding protein-related//subfamily not named
*Prupe.7G234800*	2.96	5.64	4.85	4.63	5.28	Auxin-responsive protein IAA15
*Prupe.7G244300*	1.95	2.26	1.49	2.36	2.15	EIN3-binding F-box protein (EBF1_2)
*Prupe.7G247500*	2.43	9.72	10.48	4.27	6.71	Auxin-responsive protein IAA (IAA)
*Prupe.8G153100*	3.58	3.21	1.60	3.40	6.01	Pathogenesis-related protein 1 (PR1)
*Prupe.8G153700*	3.69	2.04	1.68	1.83	2.33	Pathogenesis-related protein 1 (PR1)
*Prupe.8G153800*	1.17	1.25	1.33	3.30	5.37	Pathogenesis-related protein 1 (PR1)
Plant–pathogen interaction						
*Prupe.1G238800*	1.64	1.55	3.24	3.47	7.63	Cleavage site for pathogenic type III effector avirulence factor Avr (AvrRpt-cleavage)
*Prupe.1G526700*	2.42	1.66	1.87	2.49	2.41	Ovarian cancer gene-2 protein-related//subfamily not named
*Prupe.2G307400*	3.23	2.62	2.88	3.33	5.42	WRKY DNA-binding domain (WRKY)
*Prupe.3G024700*	4.91	5.19	3.63	8.49	8.42	Receptor-like protein 3-related
*Prupe.3G098100*	4.69	3.80	3.00	4.42	8.17	WRKY transcription factor 40-related
*Prupe.3G193600*	4.87	1.46	1.87	2.91	1.64	Heavy-metal-associated domain-containing protein
*Prupe.5G117000*	2.42	2.36	3.17	3.62	4.78	WRKY DNA-binding domain (WRKY)
*Prupe.6G286000*	1.73	1.61	2.19	3.10	4.76	WRKY transcription factor 33 (WRKY33)
*Prupe.7G091900*	1.39	1.60	1.05	1.95	1.46	F26K24.5 protein
*Prupe.8G153100*	3.58	3.21	1.60	3.40	6.01	Pathogenesis-related protein 1 (PR1)
*Prupe.8G153700*	3.69	2.04	1.68	1.83	2.33	Pathogenesis-related protein 1 (PR1)
*Prupe.8G153800*	1.17	1.25	1.33	3.30	5.37	Pathogenesis-related protein 1 (PR1)
Diterpenoid biosynthesis						
*Prupe.1G111900*	1.42	3.21	3.61	3.35	4.89	Gibberellin 2-beta-dioxygenase 4
*Prupe.1G442300*	3.57	4.13	4.05	6.39	5.95	Oxidoreductase, 2og-fe ii oxygenase family protein//subfamily not named
*Prupe.2G108400*	3.32	3.39	3.58	5.77	6.08	Ent-kaurenoic acid hydroxylase (KAO)
Arachidonic acid metabolism						
*Prupe.1G007300*	3.80	5.18	5.71	11.97	12.66	Prostaglandin-E synthase/Prostaglandin-H(2) E-isomerase
*Prupe.8G244300*	1.40	1.54	1.30	1.82	1.49	Glutathione peroxidase 4-related

Note: Function description data was from the reference genome Peach v2.0.a1 (v2.1) [29]

**Table 2:** Functional classification of downregulated genes.

Gene ID	Fold Change					Function Description
	log2(HJML 3 d/HJML 0 d)	log2(HJML 3 d/XC 3 d)	log2(HJML 6 d/XC 6 d)	log2(HJML 3 d/HY 3 d)	log2(HJML 6 d/HY 6 d)	
Photosynthesis						
*Prupe.1G482000*	−2.80	−1.03	−1.36	−2.02	−2.14	F-type H+-transporting ATPase subunit b (ATPF0B, atpF)
*Prupe.1G573600*	−4.33	−1.47	−2.20	−1.82	−3.12	Ferredoxin-1, chloroplastic-related
*Prupe.3G179400*	−4.92	−2.08	−2.95	−4.33	−5.40	Oxygen-evolving enhancer protein 2-1, Chloroplastic-related
*Prupe.4G105600*	−5.28	−1.86	−3.13	−3.61	−4.46	Photosystem I subunit III (psaF)
*Prupe.5G199500*	−1.17	−1.48	−1.70	−2.27	−2.42	Plant transposase (Ptta/En/Spm family) (Transposase_24)
*Prupe.7G145900*	−5.69	−1.91	−4.66	−3.59	−5.93	Photosystem II oxygen-evolving enhancer protein 1 (psbO)
*Prupe.7G169200*	−4.47	−1.56	−1.31	−2.24	−2.64	Ferredoxin--NADP(+) reductase//NADPH dehydrogenase/NADPH diaphorase
Endocytosis						
*Prupe.1G406800*	−2.44	−1.30	−1.03	−1.97	−1.08	Phd finger protein alfin-like 5
*Prupe.1G522100*	−1.79	−1.75	−3.26	−1.12	−2.67	F3F9.15
*Prupe.3G236100*	−2.71	−1.50	−2.10	−1.41	−1.81	Regulator of VPS4 activity in the mvb pathway protein
*Prupe.4G257600*	−2.53	−1.65	−3.72	−1.89	−3.40	Dehydration-responsive protein rd22
*Prupe.5G203900*	−5.46	−1.82	−1.84	−2.32	−2.54	Glucan endo-1,3-beta-glucosidase-like protein 2-related
*Prupe.6G006200*	−4.89	−1.78	−1.93	−1.47	−2.64	F-box domain (F-box)//Kelch motif (Kelch_1)
*Prupe.7G081600*	−3.32	−2.10	−2.40	−1.98	−2.77	Speckle-type POZ protein SPOP and related proteins with TRAF, MATH, and BTB/POZ domains
*Prupe.7G107400*	−2.46	−2.87	−5.20	−1.72	−4.71	Mediator of RNA polymerase ii transcription subunit 37c-related
*Prupe.7G107600*	−1.77	−1.77	−3.41	−1.17	−3.19	Heat shock protein 70KDa//subfamily not named
*Prupe.7G107700*	−2.45	−2.92	−5.52	−2.84	−6.52	Heat shock protein 70kda//subfamily not named
*Prupe.7G175400*	−2.52	−1.51	−1.99	−2.08	−2.71	Phospholipase D delta
*Prupe.7G204200*	−1.24	−3.32	−3.49	−2.38	−3.08	Cotton fiber-expressed protein (DUF761)
*Prupe.7G210500*	−3.32	−2.48	−2.47	−3.82	−5.23	Auxin-responsive protein-related
*Prupe.7G220100*	−3.53	−1.45	−2.60	−1.59	−3.18	Genomic DNA, chromosome 3, p1 clone: mil23-related
*Prupe.8G099700*	−3.76	−2.70	−2.23	−2.69	−2.65	BURP domain (BURP)
*Prupe.8G134100*	−3.15	−1.39	−1.96	−2.16	−3.08	Upf0041 brain protein 44-related//subfamily not named
Linoleic acid metabolism						
*Prupe.1G011400*	−4.12	−1.37	−2.45	−1.80	−3.03	Linoleate 9s-lipoxygenase 5, chloroplastic
*Prupe.1G232400*	−2.12	−2.05	−1.96	−1.78	−2.02	Lipoxygenase 6, chloroplastic
*Prupe.2G005300*	−3.16	−4.54	−6.01	−5.98	−7.13	Linoleate 13S-lipoxygenase/Lipoxidase
Circadian rhythm-plant						
*BGI_novel_G000425*	−2.11	−1.45	−1.30	−1.10	−1.19	New transcript
*Prupe.1G074900*	−2.94	−1.89	−1.18	−1.82	−2.10	CCT motif (CCT)
*Prupe.1G219800*	−1.31	−1.33	−1.31	−1.05	−1.37	Zinc finger protein constans-like 14-related
*Prupe.1G398700*	−1.79	−1.88	−1.92	−2.46	−2.86	GATA-4/5/6 transcription factors
*Prupe.2G009100*	−1.50	−1.21	−2.17	−1.47	−2.33	Helix-loop-helix DNA-binding domain (HLH)
*Prupe.2G314800*	−2.83	−1.62	−2.28	−1.75	−2.91	Dof domain, zinc finger (zf-Dof)
*Prupe.5G221000*	−2.30	−2.86	−2.80	−2.91	−3.14	B-box zinc finger (zf-B_box)//CCT motif (CCT)
Citrate cycle (TCA cycle)						
*Prupe.3G230200*	−1.93	−1.07	−2.16	−1.71	−2.35	Vesicle-associated protein 4-1
*Prupe.4G004600*	−3.18	−1.85	−1.37	−1.77	−1.67	Oligopeptide transporter 3
*Prupe.6G210900*	−1.28	−2.13	−1.49	−2.02	−2.20	phosphoenolpyruvate carboxykinase (ATP) (E4.1.1.49, pckA)
*Prupe.8G074200*	−1.34	−1.26	−1.58	−1.52	−1.93	Oligopeptide transporter 4

Note: Function description data was from the reference genome Peach v2.0.a1 (v2.1) [29]

## Data Availability

The datasets generated and analyzed during the current study are available in the National Center for Biotechnology Information database with the accession number of PRJNA954126.

## Supplementary Materials

The following supporting information can be downloaded at https://www.mdpi.com/article/10.3390/foods12081648/s1, Supplementary File S1: Statistics of clean reads; Supplementary File S2: qRT-PCR primers for candidate genes; Supplementary File S3: A total of 159 upregulated genes identified by Venn diagram analysis; Supplementary File S4: A total of 365 downregulated genes identified by Venn diagram analysis; Supplementary File S5: Gene ontology classification of differentially expressed genes from upregulated and downregulated candidate genes by Venn diagram analysis; Supplementary File S6: BGI_novel sequences; Supplementary File S7: The FPKM and functional description of candidate genes; Supplementary File S8: The FPKM of 20 candidate genes in Figure 7.
