# Supplementary material for: Transcriptomic Analysis Reveals Genes Associated with the Regulation of Peach Fruit Softening and Senescence during Storage
Source: Foods. 2023 Apr 14;12(8):1648. doi: 10.3390/foods12081648 (PMC10137801; doi:10.3390/foods12081648)
Supplement: Supplementary file 1 [file foods-12-01648-s001.zip › Supplementary File 1.pdf]

Supplementary file S1 Statistics of clean reads

Sample	Total Clean Reads (M)	Total Clean Bases(Gb)	Genome mapping ratio (%)	Gene mapping ratio (%)	Clean Reads Q20(%)	Clean Reads Q30(%)
HJML1	69.7	6.97	96.35	71.14	97.9	90.96
HJML2	67.07	6.71	96.47	71.11	97.8	90.7
HJML3	69.47	6.95	96.46	71.49	97.85	90.88
HJML4	66.82	6.68	95.32	69.76	97.79	90.69
HJML5	66.31	6.63	95.06	69.94	97.84	90.82
HJML6	69.43	6.94	95.26	70.02	97.78	90.65
HJML7	67.12	6.71	85.49	63.38	97.92	91.11
HJML8	68.57	6.86	84.89	63.03	97.94	91.13
HJML9	67.32	6.73	85.27	63.25	97.92	91.06
HY1	69.52	6.95	93.55	69.9	97.9	90.91
HY2	71.82	7.18	93.22	70.13	97.9	90.94
HY3	69.68	6.97	93.28	69.63	97.88	90.81
HY4	71.85	7.19	95.97	71.39	97.86	90.81
HY5	69.57	6.96	95.98	71.28	97.95	91.12
HY6	66.99	6.7	96.08	71.72	97.88	90.87
HY7	69.65	6.96	90.45	67.67	97.94	91.02
HY8	67.1	6.71	90.31	67.52	97.88	90.89
HY9	69.46	6.95	91.97	68.7	97.92	91.03
XC1	69.49	6.95	96.09	72.04	97.79	90.66
XC2	69.5	6.95	95.91	71.97	97.87	90.91
XC3	67.29	6.73	95.95	72.33	97.9	90.98
XC4	69.56	6.96	95.94	71.01	97.85	90.84
XC5	69.48	6.95	96.19	70.91	97.74	90.47
XC6	69.64	6.96	96.18	70.89	97.77	90.55
XC7	69.55	6.96	96.43	71.3	97.78	90.62
XC8	69.62	6.96	96.17	71.03	97.83	90.76
XC9	67.12	6.71	96.29	71.36	97.85	90.87
XCT1	65.4	6.54	96.24	72.23	98.08	91.72
XCT2	65.26	6.53	96.32	71.8	98	91.43
XCT3	63.04	6.3	96.33	71.7	98.11	91.8
XCT4	64.9	6.49	96.58	72.1	98.08	91.68
XCT5	64.95	6.5	96.43	71.69	98.11	91.72
XCT6	65.04	6.5	96.58	71.97	98.11	91.78
XCT7	65.17	6.52	96.57	71.53	98.18	91.97
XCT8	65.3	6.53	96.45	71.62	98.14	91.81
XCT9	62.84	6.28	96.31	71.52	98.22	92.14

Note: HJML 0 d consist of HJML1-3, HJML 3 d consist of HJML4-6, HJML 6 d consist of HJML7-9. HY 0 d consist of HY1-3, HY 3 d consist of HY4-6, HY 6 d consist of HY7-9. XC 0 d consist of XC1-3, XC 3 d consist of XC4-6, XC 6 d consist of XC 7-9.

XC 0 d consist of XCT1-3, XC 4 d Control consist of XCT4-6, XC 4 d NAA consist of XCT 7-9.
