# Supplementary material for: Transcriptomic Analysis Reveals Genes Associated with the Regulation of Peach Fruit Softening and Senescence during Storage
Source: Foods. 2023 Apr 14;12(8):1648. doi: 10.3390/foods12081648 (PMC10137801; doi:10.3390/foods12081648)
Supplement: Supplementary file 1 [file foods-12-01648-s001.zip › Supplementary File 2.pdf]

Supplementary file S2 qRT-PCR primers for candidate genes

Gene ID	Sense Primer（5’-3’）	Anti-sense Primer（5’-3’）
Prupe.1G034300	GTTGGCCGAGCAAAATCGAG	GACATTGCTCGTCCTCACCA
Prupe.2G176900	GGCAAGGTTCCTGGAGACAA	CACAATCACACGCCAAAGCA
Prupe.3G024700	CAGCCACTTACCGGTGTTCT	GTGAGGGAAGTGTGATCGCT
Prupe.3G098100	AGCCTGGTTGTGAAGGATGG	AGCTTGAGGAGGGGACTGAT
Prupe.6G226100	TTCTTGAGGTCACCGGCTTC	CTCGTGTACTCAACCACGCT
Prupe.7G234800	ATGGAGCTCCTTACTTGCGG	TAAGCTGCTCCTGGTTGCTC
Prupe.7G247500	AACACTCGCAGACACCTTGA	GCAAGAAGCCAGTCTCCCTC
TEF2	GGTGTGACGATGAAGAGTGATG	TGAAGGAGAGGGAAGGTGAAAG
