# Supplementary material for: Transcriptomic Analysis Reveals Genes Associated with the Regulation of Peach Fruit Softening and Senescence during Storage
Source: Foods. 2023 Apr 14;12(8):1648. doi: 10.3390/foods12081648 (PMC10137801; doi:10.3390/foods12081648)
Supplement: Supplementary file 1 [file foods-12-01648-s001.zip › Supplementary File 5.pdf]

Supplementary file S5: Gene Ontology classification of differentially expressed genes from up-regulated and down-regulated candidate genes by Venn diagram analysis.

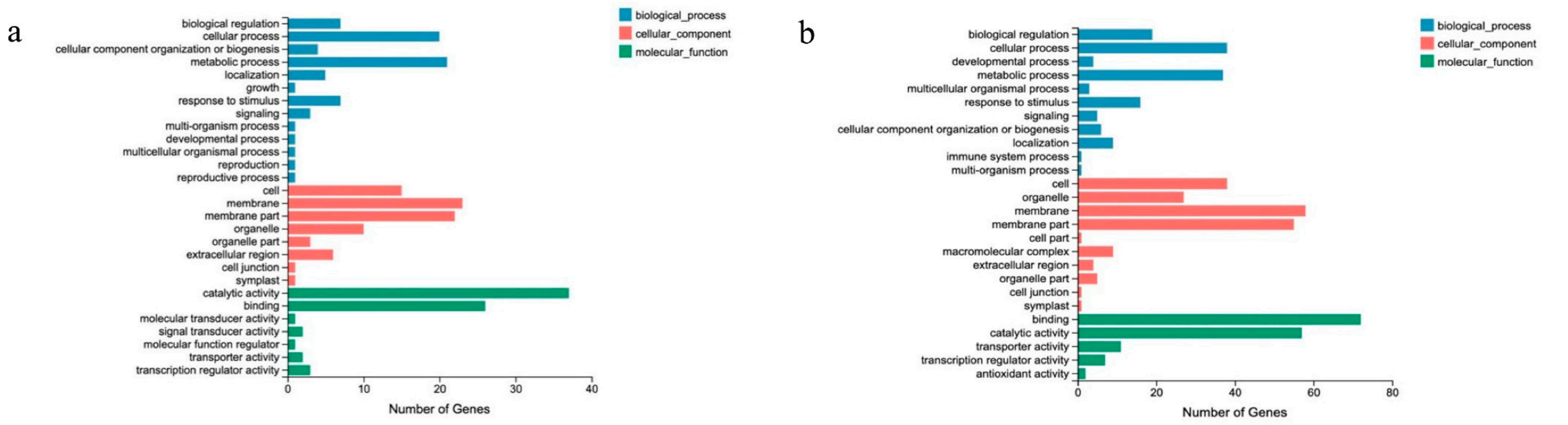

a, Gene Ontology classification of 159 DEGs from up-regulated candidate genes. b, Gene Ontology classification of 365 DEGs from down-regulated candidate genes.
