# Supplementary material for: Transcriptomic Analysis Reveals Genes Associated with the Regulation of Peach Fruit Softening and Senescence during Storage
Source: Foods. 2023 Apr 14;12(8):1648. doi: 10.3390/foods12081648 (PMC10137801; doi:10.3390/foods12081648)
Supplement: Supplementary file 1 [file foods-12-01648-s001.zip › Supplementary File 6.pdf]

Supplementary file S6, BGI_novel sequences are as follows

>BGI_novel_G000319, Pp02:24127171-24128744 -
GTAAAAACTCAACTTCAAGAAAAACATACAAATTAAAAAAGCCACACAATTCCTTCTCCCAATGTAAT
CAACAATATGTTTCTATAGAACACAACTTCACTGAATTAAAAATAAATAAACAAGCGAACTCTCCAAAA
TGATCAAACAATTGGGACAGTGTCGAGGCCTAACACCACCAAAAGTATTGAATTTAATACTGAGTTGG
CAAGTAGGACTTCATCATTCACACCACCAAAACCTTTGCCCATCTCACCCAACAGAGGGTCAAGATCA
TTCCACGTGTCACACCCATATCAAAAGAAACCCGAATTTCCCTCACTTTTTTCTACCCCTAATTAAACCT
TCTTATAAATATACAACCTGCCCCATGCAAATACACCAACCTCCTCTCTCTCCCTAAAAAAACCATCCAT
GGAAAACCGCCGGCAAAAGAGCACCTCCGGCGACACATTCTCCTTTCCAAGCACTTCGATCCACTAG
CAAGACTCCGATTTCGAATTTGGATGCTTTACCCCTGACTCAGCCTCCAGCACTGATCCCTGCAAAGA
CTCCCCGGCAGACCATTTGTTCTTCAATGGCAGGCTCTTACCACATGCCTTTCCATGTCAACCAATTAG
CAACGGTTTTATGGGTTTCGATAGTAATTTACGGAGAACAAGCCGGATGAGCAGCATTAGCAGCAAGG
ACTCTCTCATGTCCTCAAGAAGCAACAGCACCAACAGCAGGAGCAGCAGCTGCAGCAGCAGCGCAA
GGACAAGCTCAAGCGACAACTCTGAGAGGAAATTGGTGCACCAAAACAAATTGGGTTCTAGAGCTTG
ATCAGATAGATACAAAGGAAACAGAGCTAATAATCTCGGAGGTGGACAAGTTTATGGTTCTTCTCAGA
GATGGCAATTCATAACAGCTGTGCCTGTACCGGCTTCTTTAAGCGGTGACAATTCTCGGAAGAAGGCG
AAAGTGGATCAGCTTGAAGGGAAGGAGAAACCCATCAAAGGGAAGGGAAGAGTCCGGGTGAAGAC
GGTGGTGGTCCCCCGGAGTTTTGGCCGGAGGTTTTTCCGGTGGCTTGTTTGGACCTGCAGAAAGTGTA
ACGCAATGGAACCGTCCAACAAAAGTAGTGATTATGTAATTAATTAAAGCTGCGAATTGATAAATGTGA
AGTGAAAAATGATTTTTTTAAACTTGTTTATCAAGTTTTGTAATTTGTTCATGGGTTTGAGGTTAGGATT
TTGAGGTTTGCTTTGAAAGTAGATAACTGACTTTATACTTTAGCTTTGGCTTTGGCTTTTGATCACTTTC
AACTTTGAAGAAAAGCAAAAGGAAATATATATATATATATATATATATTCTGGGAACGTTAACCAGCAAA
AGCAATTTGGCTTGTTAAAATAGAGCTTTTGTCACAATGTGTTCGGTTATTGTTTCAAATTCTTCAGAGC
GAGCGTTTAATCTTGAAGTTGAAGCTGTTGATATGGCAATGATATTCTTCCATAAATCCTTTGGAAACAT
AAACTAGTTGGTTGACTGAATAGTGATCAGTCAACCAAATCTTTATCTAAAAGCCAAGGCTTGTCGTG

>BGI_novel_G000473, Pp04:12563396-12565812 -
TCTATTTTCACGCTCTCTCTTCCTCCTTCCCTCACATCACGTTGCTTCTTCTCACAATCTCTTCACAGAG
CCCACTCCCCTCCTTCTCTTCAATTCTGCCTACAGTAGTTTTTTCTTGTTGGATCAATTGAAGCGCATTG
CTGAGCCACAAGACTCAGAAAGGGTATACCTACCCAAAATGTGATGGCTGCATGCAATTTATATTTGCA
TGTGCCGGATGGGAAGGCACGGCCCATGACACAAGGATATTTCTATCGGCTTTACGTGATCAAAGTTT
GAATTTTCCTAAACTTCCAAATGGAAAATTTTATCTAGTAGATGCGGGATACCCACAAATGAGAGGCTA
CTTAGGACCATATAAAGGTGAAAGATATCATCATCTTCCTGATTTTCGTAGGGGTGTTCAACCAATGGG
TCATAAAGAGGTATTCAATCATACACACTCTTCTCTGAGAAGTGTTATTGAACGCACTTTTGGAGTATG
GAAAAAGAGATGGAGTATTTTACGCGACATGCCTAGTTATCCTTTTGCAAAACAAGTCAAAATAGTTAT
TGCAACCATGACACTTCATAACTATATAAGAAGACATGCCAAACGTGATTGTCATTTTGATGAAAGTGA
AGATGACCTAAATGGTACTCAAGATGAAGAGATTGAGAGAGAGGTTGATGCACAACAAGAAGATGGT
CCTGTAACACAAGAAATGGAGGCATTGAGAAATCATATAACCGCAAGTTTAATGAGTTCATCTGGTTAG
AAATGTATATGATTGCTTATTTATGACTAGTTTAATTTTTGTTATGACCATATTATTGACAATATATTTATTT
TTAATTGAATACTTTATTTATTTTATAATTAAAA

>BGI_novel_G000666, Pp06:15003576-15004186 .
CAATTTCTTCTTCCATCACTAACAAAGATGACCCAAAAACCCTAGAGAAGTGTAAATTTGAAACCACA
AGAAGATAAAGCTTTTATGCAGAGGTTGAGCGTAGATTAGTGAAGATGGTGTTATTGGAAAAATTCAAT

CAAATAAAAACATTTTGGAATTTTGTCTACCAAAAGTTTATATCCCGACAGTGCTGCCCCGGATCTCAA
ACACCTCAAGCCATTGCATCTCAAATCAAGACCATCAACCAACAATACTCTCTATGGAAGAAGAGAAG
TTTGAAATAAAGGCAACTCAAACTCAATAAGTGGCTCAAATCTTGGTCATGCAGTGAGTGCCAATTCT
TCATATCTTAACGTTGTATTCATAATTATATTTATGAACCAACTTATTTTTCCTTTAACCCATGTATAGTGC
TTTCAAGCAAATTCATATTTTTGAATTATAATAGGCTTCTAAATAGATCTTTTGAGTTGTATCATGCTTGA
CACATCTTAAAATATTGCTCAAAATGGAACGATCAATTTCAATCTCCTATGGGAAGATCATCTTAGCATA
GTAAGGAGACCATTAATTCAAAGGATGATGCAAAAGATGTGGTAATGGCATATTCC

>BGI_novel_G000725, Pp06:27354244-27359524 +
ATGGAATTCACATGCTTGTAAGGTGTCATCCGCTCCAAACAAGATCAAATTCAATGCATATTATCTAAAA
AGATGATGAAAGCCACAAGAAAAGGAAGAAAAAGAGAAAACAATTCAACAATGGAATTATGGGCAA
CCACACCAACATAGACAAGATGAAGTGGGAGTTATGGATTGTATTAAAATAGGAATGGTGGTGTTGGA
ATTCATCATAATGTTCCTCCCTCTTAGCCCTATAAACAGAGAAGTCTTCAAGCTTCAAAACACACACCA
AGCTCACAATCTTCTCTTGAGCTCTCTCTCCTTCCTCCAATTCTTCTAGTTCTAAATTTCTGTTTTAGTTT
TATTTTTTGGGGAACTAACTCCCTAGCTAAGGCTAAGGGGGAAGCTTTTAATATGAGTATGTAATTTTTA
TTAATTTGATCCAAACTTAAGCTTTAATGTGAATTTGTAGTTTTGTTCAAATGGAATTTGTAGTTTTTCTT
TTTAATTAATTGATGAATGTTTGATAATCTATTTCATGGGTTCAATAATGTATTTCCCATTGTGTAGTAAAA
ACTTTCATGCTTTTAAAAGATGAGACAATTACTAAGATAGATAGAATTTCGATGAAGATTATTTTTTGAT
AGTTTGTTGCCAATGGTACAAGTTATGCTTAGAATACTTCTAAAGGAAATTAAAAATAGTTTTAATTATT
TTGAACAACTACAATGGAATGGATCAAATTAATAAGAGTTGCATGCTTATGTTAAAAGCGGAACCCCA
AAGCCTTAATGTTTTAATCAATTTAATTTTCTTTAAATCAAATCTTGCATAAATCTACTTCTCAATCTTAAT
TGTTCTATCTCAACTCTGTTATTTTCTTTACACTACTCTCTGTGGATACCACACTTGGACTTCCAAACTA
CACTGCTATACGACACCTTCACTTGGGTTTTGCAAAGTGGTACAAATCAATTGGCGCCGTTGTTCGGG
AGCGGCTTGTCAAAGAGTACAGAGATCCCCTTGGTTGGAGGTTTGCTGCTTATGTGAAATTCTTCACAT
CCGGAAATGAAAGGAGCTTATGAGGAGCGTTGTGCTGCTATTGGAAACTTCAAACAATAGTATTCTCC
TTTTTCCAACACTTATAATCCAGCAACAAGGAATCATCCAAACTTTTGTTGGAGCAATGAGTCACAACA
TCAGAAGCAAAATCAAAGGTATGATACTTTGCTTGGTCAATTGGGTGAAGAGAATAGGGAAATTGGCA
GCTTTACCCCTGGCTCACCTAATTTATTCACTAATATCCTTATTTGAAACTCAATCACAAATGCCCCTTA
TAAAAACTCCTTATTGACAGCCTATTGCCCATCCTTCACTTCGTTTTTTTTCCTTAGTTGTTGCTCTTCCT
CTTCCCCTTCCTTTTCTTCCTTTTCTTCCTTCTGCCAAACCTCACAAAATCCACTTCAACCATAGAATCT
CCCTAAACTTTATCTCATCTTAGTTATATCTTCCAACTAGGAGCTGATTTCAACCTCAAAGTTCAACCCC
AAATTTGGACCGGACCCTAGCCAAGGCGCGACTCTCCACCTCCGTTGTCGGCCTCCACAACAAAGCC
ATAGTCTTGACGTGCCACCTCCATTGTCGACTTCCACCACAAAGCCACGGCACCTTCCTTGTCGATCTT
CACTGTGAAATGGACACAATTGCAATTTTGGTTTGCTACAATGGCAAATGGGTAACCTCAAAGAAGAT
GTGCACATATGAAGGGGGTGACTCAAAAGGCATAATAGTTTCGCAAAACATCACGTTTGGTGAGCTTT
TGGAACGGGTGCATAAGATTGTTAATAGAGACAACAGGGAAGACAAGCTTTGTTTAAAGTTCTCAATT
TCAATATCCTCGAATGAGTGTAAGCATATCAAGATTGAGGACAATGATGATGTTTAATTTTTTTATTAAG
TACATTTGTGAAGTAATTCCTTCAAAAGTGGCTTCTTTACTGGTGAGCATAGAAGACAGAGGAGTGAC
AAATGTTGTAGGTGATCGTATACATATCACGACGGATATGAGTTGGATGGCCTGTTCTTCAAATGCCATA
GTTGAAAGCAATGGTGATGTACATGGTGATTTTGGAACAGAATATTTAGATCTGATTGATTTTAAAAGG
GTGGAGGGGATGCATAGTGGTGATAATGGTACGGAAATGAATGGCTTGCAAATGCACTCAGCCCATCC
TATTTTGGCCCATTTGGAGACATTTTCACAAGTGGGTGTGAGGGGTTTTGGAATAACGTCTGAACTTTC
TATTCGACATAATTAGAGGCAAATGGGTGAACAAAACGTTGACAATTTGGGTATTGTAAATGATGAAGA
AGAAGATATTGAAAGTGCGCATTCACGGAATAATTGGGATCCAAAATTGGAAGTTGGGAAAATTTTCT

CTAGTAAGGAAGCCCTGTCCAACAAGTTACAGTTGGTGGCTGTGAGAGGTCACTTTGAATTTAAGGTG
AAACAGTCTTGCAAGAGTTGATTAGTTGTGGTTTGTTCTCAAGGCCCATGCCCATGACGGCTTCGTGC
ATCTAGTTATGGAGAAAAGAACTTCACGATTGTGAAATATAATCCAGTTCATGAATGTGATATGAGTTTT
ATACATGACAAGCATCGTCACGCAAGTGCTAAACTCGTTGGTAATGCAGTGAAGCAGAAGTTCAAAG
ATTCTCGCACAATATACACAGCAAGTGATATAATCAAAGATGTGAAACAAAACTTTGGTGTCCCCATTA
ATTATTGCAAAGCTTGGAGATCGAGGGAGTTAGCTCTAATATCCATTAGAGGTTCAGTAGAGGAGGCAT
ATTCTCTCCTTCCAGCTTATTGTCATGAATTAGAGCGTGTGAATTCCAGCACAAGGACATACATCCACA
CCGATGAGAACAATCACTTTTTGTATTTTTTATGGTTGTTGGGGCATGTATTAGAGGGTTTCAATCTTTG
AAGCGGCTAGTCATTGCTGTGGATGCCACGCATTTAAAATGTAAATATAAGGGTGTCCTTTTTGTTGCC
AATGCATTTGACGGAAATCGAAATATATATCCTCTTGCTTTTGGAATTGGGGATTTAGAGACGGATGCAT
CATGGCATTGGTTTTTCAGTAAGCTTCATGAAGTCATTGGTGAGTGTCCAAATCTTGTGATTATTTCTAA
TCGGAATATTAGCATCGAGAATGTGTGGCATAACATTTTTCCAAGGCGCAACATGGCATATGCTTTTACC
ATATGAAGAGGAACATGAAACGCACTTTCAAATTGAAAAAAACGTGATAAATTATTGCTACACTTTGA
ACAAGTTTCAAAATCTTATGGCATCGCTGAATTTGATCGTCATTTTCGCAAGATCAAGGGAAATGATGA
GGTTGCTCAATATCTTGAAAGTGCAGGATTACACAAGTGGTCTAGAGCTCATAGGG

>BGI_novel_G000067, Pp01:20390408-20393408 +
CCTCAATTCATTATTTGAGGTTTATATTGATATTATCCATTTCGCGGTGTATTCTTAACAACCGGAATTCA
CAAAATATATTTCTTCCTTAAGGTGTCGATTATAACAAAATCGAACTTTATTAAATTCATCATCTTCTTAT
GCCAAAGAAATATGTGGCGTACCACAATTTGCAATAATACCTCAAGGGTTGTCCATTTAATTGTTGGAA
CTTCAGGTTCTCAACACTGTTAGATTTTGAACTTCAGGCCAAAATCATATATTCTCATGGTATGGACATT
TTTACAATTTTCTGTACATATTTCGGGACTTCAAGCCCTTACATAATTGTCCATATTTTGAGGAACTTCTG
GCATCTCATTTAATTGCTCATCCATGAGTTTAAGAAACTGCAGGTTCCCTTTTGTATATAGTGACGGTTT
ACCCAAAATGGTAAATATTTATGCATACATCACTATTCATGTATACGGCACTATTCATATGTACAGTACAT
TTGCCAGTACAGTTATCATCCATGTGTACGGCATTATGAACCAATACGGTACTGTTACATCATTAAGGAA
CCCCAGGTCCTTATTTACATGTCAAGGATCAAGGACCCTCAAGTCCGATCACATGTTTACAAATATAGT
GCCGGAGAGACTGCCAGCTCTCATATTAACATCATCATCAAGGATCTTCAAGTCCTGATGTAATTGTATG
ATGAGGATCAAGGAACTTCTGGTCCTGATCTGCATACTGTAAAAAATTCATCATACAGCACATTTAATC
CATATAAGAAATTGCTGGTAAATAAATTACCGGTATGGACGATAAACCCGCACCATACATTTAAAAGTA
AAGGCGTGGACGATAAACCCGCACCACCCTTTAAATAAATACTGTTGTATGGGTAATAAACCTACACCA
TACAGTAAATAAAATAAATGTGCGGTAAAGTAAATTTCATAAAGTAAAGTGTTAGTATTAGTAACGGTC
TCCCACTGATAATATGTTTAGTATTACCGAGGGTCCATTTTTGTTAATGGTTAGTAAAAGAAAAGCAGAT
AAATATAATAGGAAATAATAGAGAAGATAAGGGAGCAGAAAACTTATCGCTTATTCCCTTGCTGAGTGA
TCCCCTTTTTTTTTTTTATTATTATTATTAATTATCTTTGCAACTGTGATCAGGCGTGGGTGGGAGAGAAA
TGCCAATCAATTTTGCTTTCCCACTTTATCTGCCAATCAATTTTGCTTTCCCACCTGCCAATCAACTTTG
CTTTCCCACTTTGTCTGCCAATCAACTTTGCTTTCCTTTATCGGTGGGTTTGCTTTTCTTTATCGGTGGG
TGGACTGTGCACATGGTTAGCGCAATAACAAAAACAGTGAAAACTCATGAAAGTCTAGGACTTTTCAG
GTCATCTTCTTCAATCAAAGAGGACCCCTTTTTCTTTTCTTTTCTTTTTTTTTTCTTTCTTTTTTTTTTTTT
TTTTATGGATCCCAAGGGCTCAAAGCAGACACTAGAGATACCCAGCTTCTTGAGCCTTCTTGGCTATTG
GAGTATCAGTCTTAATTTTCATGTCAGGCATAGCGGGCATGGCACAAGAGGCGACGGGCTTTGATGGA
GGCTGGTGGGTCTGAAAAACAAGAGAAAGCCGGCACTGCTGCTGACCAAGTCGCCGGACCCAAACC
TCTCCAGGACTACGAGCTCTTCGATCAGATCGGGTCGGCAGGACCGAGTCTGGTCTGAAAACTGTACT
CGGCCAAGGCACTTTCGGCCTCCTGAATCTCGATTGGGCCACCTCAGTGCCTCCTGATTCTTCAGCTCC
TTCGAAACCTATCATGCCAAGATGATGACTTGACGATTTTAATGGTTTCAGTGCAGCAGTAGAGTCTTG

GTGGTATAGCACACAATCCTCTCGATCAAGACCACACAATCTCAAATAAGGCTCAGCAGTCACCGATT
GAAGGCAACAGAATTTGATCTTCCTGTACTTGTCTGCTTTAATCACCCACTTCACCCCTCCATGGCAGA
GATGGGAGACCCCATCGTAGACCACAACGCGTGGCTGAAGAAGAGTGGGTATCACGGGCTTAACCGC
CGAATCAGCAGTTGGGTTAGAGTGGATGGGTACATCATCTTCTTCTCCTCCATATGAATTAAATACTGTA
GGAACACGTGCACAGCCCTGGTATGAAGTGGGAGGGAGCTAGCTGGCCGCCGGAGATGAATTCGGCT
GTGCTAAGACGGGTTGATGGTGTTGTCGTAGCTGATCAAACCCCAAACCTTCATTGGATCAATTAGCCT
CTTGTGGTAGTTCGCCATCTGGGTGTGCAGAGGAAGCTACTGGGTGTGAATAGTGATTGTTTGGCGGG
TGTTGATCTCGTCAGCTAGCTTTGTTTGGGTCAAGAAGCTGTTATACGACGACGACGGTTTGCTTTTCA
ATATTGGGTTTGGAAAAGTGGTCGTTGTTGATAACCTTCCGATTGTGAAGGCGGAGATGGCCCAGCAG
CTCGAAAGAGAAATCCGACGAGTGTTTAGTAAAGTTGGTGTTTGATGAAATGAGGGAGAGAGATTTG
GTTTTGGTGAATTTTGGCAGGGAATCAATCACATGCTCTCTGTGAAAGAAACAGAGCTTATGTTTTTCT
TGGCTGGGTTTCAACATAGAATTCAGCTGGAATTGTTGAAAGCAAATTCGTGCTGATAACGTGTTATAA
AACTGGAGAAGTTTGGAGAGAATTGAGAGAGAGAGAGGAGGAGAAACTGATTTTTTTCTTCTCATTC
AGATATATTCCTGATTATGAAGGGATGAACCCTTATATAAGCAAAGCCTAAGAAACATGTGGGCTCCAC
AACTAATTATTTACAACAAAAAATTAATCCCGTTTAGACAAAATCCGTTGACAGGATTATCATTGCATAA
AGCAAGTCCCTAAAGGACATGTGCTTGAAGCACAAAATTTGTACTCAATATTAATATGCATAAATTACC
TCAGTGAGTACATTGATGTGATTGCAACA

>BGI_novel_G000071, Pp01:20617419-20617829 .
TTTAATTTATGAATTTAATTTACCGCACATTTATATTTATTTAAAAGGGTGGTGCGGGTTTATCGTCCACG
CCTTTACTTTTATTTAAATGTATGGAGCAGAATTAATGCCCATACAAGCACCTTAACTTTATGAATTTAAT
TTACCGCACATTTACATTTATTTAAAGTGTGGTGCGGGTTTATCGTCCATACCAGTAATTTATTTACCAGC
AATTTATTTTATGGACTAAATGTGCTGTATGATGAGTTTTTACAATATGCAGATCAGGACCAGAAGTTCC
TTGATCCTCATCATACAATTACATCAGGACTTGAAGATCCTTGATGATGATGTTAATATGAGAGCTGGCA
GTCTCTCCGGTACTATATTTGTAAACATGTAATTGGACTTGAGGGTCCTTGATCCCTG

>BGI_novel_G000123, Pp01:37238831-37239585 +
GCCCTATATAAATGGGCTCCTTCATAGTGTGCAAGTCACCCAATTCAAGCCTTTGTTAATTCCTTGGTAG
TTTTTGCAGTTAATAAATTAGTAGAAGATGGGTTATCACAATAATCTTGATAAGGTCCGTAATAAGAGGG
CCAAGTTTCCCTACACAAAGGAGCAGATAAGGAACGTCTTCAAACGTCATGACAAGAATGGAGATGG
TCAACTTTCCAAAGATGAGCTGGACGCAGCCTTCAAAGAACTTGGCTCGATATGGCCTCCTGGTAGAG
CTTGGTTTGCTCAAAGGTATGCAGATGACGACGGCGATGGCTTCATTTCTATAGACAAGGAGCTCAGC
AAGCTCGTCCAGTATGCCCTAGAATTGAAGTATACACTTAAGTAATTATAGTATTAGTGGGTAACTAGTT
CTTGTATGTGCTTGCTATAGATATATTCTCAGCAAGCTCGTTGACTATGCCCTCAATTGAAGTTCTCTATA
TAAGTAATCGTAGTGGTGGAGCTAAACTTGGTGTCTTAGTGACCACAACCACTTCAAGTCTATATGTAA
CTCCGTCTGTGTGTAAGCGTCTTTAATTACTTGTAAGCAGCCGTAGTACCTATATATGTGTGCAAGGTTG
AAGGAACGAAAGAGGTGACCTTTTGTTTCCCTTCGTTACCAGGGAAGATGAATTTGAACCCTGATTAT
GTATAATATATAGTGATTAACAAGAATAAAGTAACAAACTTTGTGTATTGATATCTATTACGTA

>BGI_novel_G000124, Pp01:38297552-38300927 -
GTGTGATTAAATGATTAAGCAAGATTAGAAGAAAGTAGATTGTGAAATAAATTGCTACTATTTTAATTCC
ACTTTTCCAATACCACCTTCATCAGGAAGTTACATTTTTTTCAAAAACAAAATAAATCAAATTGTGTTG
TAAAAGTAGAGTCTGGAGTCCCCAAAATGCCCCTCCCCTAATGTCTATAAATTGGACTTCCCTTAGATA
GAAATTACACAATCTGAGATGAATGAGAAATGCAAATAAAGAATTCAACAAGAATTCAGATTTCTATTC

CTTTCTCTCTCTCTCAATTTCCTCCTCAATTCTCCAGTTTTATAACACGTTATCAGCACGAATTCTCTTTC
AAAAATACCAGCTGAATTATCTGATTTTTCTCTGCTTCTGGTTTCTTCTCTCTGTCTCTTCTTTGTTTTGT
GTCTCAGAGGCACAACAACACCAAATGGGTGTTCTTAAGCAATTGATCTCCATCTTCTTCCCCCTTGCG
GTTTTGCTAATCACACCCACAACCCTGCAAGATGGCCAAAATGTCATAAAATATATCAAGAAGAGCAG
CAAGCCCCCGGTTCATTTGTCCTGGCCTCATGGTCTCCAAATATTTCAGTATTTGAAGTATGTATATTCAT
GAGAAGGAAGCTGGGTCTGAAGTATGTTAGTGCGTTTTCTCCCTTTGGGAGTTTATGGAGCTTCCTCCT
CGGCTACCAATACCGGGATAGATTATTATACCATTATTGAGGACCCCGCTGCTGCCAAAGGGAAGACTC
TGCCACAGATCATACTGAGATCCATCATCCAGCAAGGAGCTTCAACAAGGAGAGGATGAAGCAGAAC
CTTAAAATATTCTATTGGGAATTAGGTGAAGATGAAATGAAGAAGATCAACCAAATTCCCCAGCGCAG
GTTGTATGCAGGAGACTTCTTTGTTTACGAGCTTGGACCTTACAAGTCCCTCCACCAACTTTGGGATGG
CGACCCCTAGGCCCTATCCTACCCTAGCTACCCAAAACTTCATGATTTTCTCTTCCAGATATGCAGACGC
AAATGGAAGCAGCAGATCCACACATCTCAATCAATTTAGCACCACAGAGCAAAAAAAAAAAAAAAAA
AAAAAAAAAAAAAAAAAAAATGAAGAAGAAAAAAAGGCTGCTTAATTGCTTTTGCTTTACAGGAAGA
GGAAAAAAAAAAAACCAAAAGAGGGAGGAAGCAACGGGTGGAGCACTCAACTCAAACTCTTTTACGT
CACTTCACCCCATCTGTCAACAAAAAGCGCTTTTTGCATTGTGGCCGGCATTATTGGTGCTGGCCAACT
TGCTTCAGGCTTAAGTTTTGAAGTTTTCTTTAAAGCCCCATTGATTGTGCCGCTATCGAAATTACGCATG
CACACTAAAGAATTGCTAGGCTTTACTGATTGACAGCCACCCACTTGGTCTCTTGGTTTTGGAATCTTA
ACGTGCACTTCGGTCGGGGTAAATATGACTTCTACTTTATAAAATGATTTAATATAATATCATACTATTTG
ATCGTGGTGTTGATGAGAACCCACCAATAATTGCGTTTACTTTATCGCAAATTTACTTTTACTTAAAGTA
TGATGAGGAATCGACCCTCATACTTTATAAATTTACTTTACTGCACATTTAATTTCATGGAGCAGAATTA
GTGCCCATACACGCACGTTTAATTTATGAATTTAAATTACCGCACAGTTACATTTATTTAAAAGGGTGGT
GCGGGTTTATCGTCCACGCCTTTACTATTATTTAAATGTATGGTGCGGGTTTATCGCCCATACCAGTAATT
TATTTACCAGCAATTTATTTTATGGATTAAATGTGTTGTATGATGAGTTTTTACAGTAAGCAGATCAGAAC
CAGAAGTTCCTTGATCCTCATCATACAATTACATCAGGACTTGAAGATCCTTGATGATGATGTTAATATG
AGAGCTGGCAGTCTCTCCGGTACTATATTTGTAAACATGTGATCGGACTTGAGGGTCCTTGATCCCTGA
CATGTAAATAAGGACCTGGAGTTCCTTAATGATGTAACAGTACCGTATTGTTTCATAATGCCGTACACAT
GGGTGATAACTGTACTGGCAAATGTACTGTACACATGAATAATGCCGTACACATTGATGATAACTGTACT
GGCAAATGTACTGGAACCTGCAGTTCCTTAAACTCATGGATGAGCAATTAAATGAGATGCCAGAAGTT
CCTCAAAATATGGACAATTATGTAAGGGCTTGAAGTCCAGAAATATGTACAGAAAATTGTAAAAATGTC
CATACCATGAGAATATGTGATTTTGGCCCGAAGTTCAAAATCTAAAGGGATTGAATGTCCATACCATGA
GAATATGTGATTTTGGCCCGATTTTGGCCCGAAGTTCAAAATCTAAGGGGATTGAATGTCCATACCATG
AGAATATGTGATTTTGGCCAGAAGTTCAAAATCTAACAGTGTTGAGAACCTGAAGTTCCAACAATTAA
ATGGACAACCCTTGAGGTATTATTGCAAATTGTGGTACGCCACATATCTCTTTGGCATAAGAGAATGAT
GAATTGAGGCTCCTTACATATTTTGTTATATTTGGGCCGGAAGCTCATGAACCCAAATATATGAGATATTA
GCGTGGCTTTTACTCAATTCATATTTCATGTCCATTTTGAAAAGGGCAAATAAATTCTGAGTTTATGTTT
AGCCCATGCCAAAAGTTCCGGGCTTCCATACGTTGAAATATGAAATTTGGGACAGTCGATGTGGTTATT
TGAACCTATAAGGCACAAGGTTCCAAACCGTCATTTTGAAAAGACGTAAAAACCACAGGTGCAAATTT
TAAGAATGTGCGCAATTATTATAAAAGGAAAGGAGCAAGTTGAAACGCAGTTGCTCCAATCAAGTTTT
GCAAAGGCCATATCTATAGGAGGAAATATGGCTACAGTTTCCAGGATCCCAATATTATCTCAGATCCCCA
TATTATCTTTTTCTTTCCCAGATTCTAAAAACTTCACGTGGCCTCCATTAAATGTTTGTCGGCACTTTCG
GCGTTTTCAAGTATTATTTTCATCAAAAGCCGATCTTGCTTTACGGATTTCACGGAGCTACTTTTTCAAA
AGTACCCCGAGAATCTCGCAATTTTCCTATGGTTAATTTGAAATTCACCGATTTTACCTATTCATCTTATT
TGTGTATAAATGTGTTACTAACGAAATTTTCTTTGCAGAAGACATCCAGTTTTCTCCATACCTAGTGATG
CGATATCTACTTGTTTTTCTTATCAGTGAGCACGCAGTGATGCAATATCTACTTATTTTCCTTATCAGTGA

GCACTTAATATGCCCGAGAAGCAAGACTATCTCTGATCATCAATTCAG

>BGI_novel_G000254, Pp02:11721719-11726435 +
CACAATATCAATATTGACATGTCAAAGGATTGTCCATTCGGGACTTCAATCCACATCATTTGCTACGCAA
CAGGCGTGCATCATATTGATCACTGCTATACCCATATTCTGTTCTTATGGGACTTTAATCTGTGCAGTGTA
TTATCAATGTATCCAACTTACAATCTGTTAAATTTGCATTAATAATTCATGGATACATACAAGCCATTCTG
TTGGAACAGACTTATCTCCCCATTTGGTTACCTTGCAAGATAAAATATCAAATAGGTATATAAGCATAAA
ATAACACAAGTATTGCTTACATCCTTAGGTGGGAGAAACTTTTCTCAAGTATCATTCAAGCGCGTATCG
GCCCAGTAAAAATATCGCTTGATGATCTTGTTATATCCTGCAAGAGCAAAAATCACAATTCTTTTCTCTG
TCAAAAACAAATAACTCTCAGAGTTAGGATCACTAAGTATAGAGAAAACTGGATGTCTTCTGCAAAGA
AAATTTCGTTAGTAACACATTTATACACAAATACAATGAATAGGTAGAACCGGTGAATTTCAAATTAAC
CATAGGAAAATTGCGAGATTCTCGGGATACTTTTGAAAAAGTAGCTCCGTGAAATCCGTAAAGCAAGA
TCGGCTTTGAAAATAATACTTGAAAACGCCGAAAGTGCCGACAGGCATTATTAGAGGCCACGTGAAGT
TTTGGACTCTAGGAAAGAAAAAGATAATATGGGGATTCGAGAAGATATTGGGGTCCTGAAAAACAGTA
GCCATATTTCCTCCTATAGATATGGCCGTTGCAAAACTTGATTGGAGCAACTGCGTTTCAACTCAAATTT
CTTCATTTTCTGAAACTTTAGTTTTCCTAAGACTTTCTTCGAAACCTTCTCAAAAATGGCTTCCTCTTCT
TCCTGCCCAAATTATTTCAATTTAAATGATGCTCCAACAGCAACCAGTGACGCCAAAGTTTGGCATCCA
TCCTTTGTATCCCAAAATCGTCATCTCACAGTTAATGATTCTGTGATGATGAATGATGCTACTGCTGTCA
TAGTAGCTAGGAATTTCATCACTCCAATGGATGAAATGCTGTTAACAGGGAGGTCTGAGGAAGAGGCT
ATTGATGACTCAATGGCTTTTAGCATTCAGAGTGCTGCTTCTGTTTCTAACATGGCTGATCGTTTGCGTA
CTAGAGCAAACGAGGTTGAGAAGCTGACAACTGAAAATTCGTCTCTTCAAAGAATGCTTCATGAGTCT
CAACAGGAGGTTGAGAAACTTAAAGGAGAGAATAATTCCTTGTTGAAACTGGTGAGTTCGTACTCAG
TTGATACACTGAGAAGGCTAGACATGCTGCAGGTCTCCAATGAAAGGATTTTGGGAGACCACGAGAA
GCTCATGGCTAAGCTTAAGAGGCGCCGTCCTCTTCCTTCAGAGGCTTCTAGAACATAATGGATTTTTAT
AGATTTTACAGGGCCTGCACCTTGATTGCAGGTGGAAAAATCTATCTGTTATATGTTCCTGTAATAATAA
TAGCGCACATTCTAAAACTTGCACCTGTGGTTTTTACGTCTTTTCAAAATGACGGTTTGGAACCTTGTG
CCTTATAGGTTCAAATAACCACATCAAATCTCTCAAATTACATATTTCCATGCATGTGAGCCCGGAACTT
TTGGCCTGGGCACAAACTCAGAATTGATTTGCCCTTTTCAAATGAAATATGAATTGAGTAAAAGCCATG
ATAATATCGCAATATAGTAGTGAAGAGCATTAACTACTATATACCCACAACTTCAAGTTCAGGATCTCTC
ATATATTTGGATCCATGGGCTTCCGGCCCAGATATAATAAAATATGTGGGGAGCCTCAATTCATTATTTGA
GGTTTATATTGATATTATCCATTTCGCGGTGTATTCTTAACAACCGGAATTCACAAAGTATATTTCTTCCT
TGAGGTGTCGATTATAACAAAATCGAACTTTATTAAATTCATCATCTTCTTATGCCAAAGAAATATGTGG
CATACCACAATTTGCAATAATACCTCAAGGGTTGTCCATTTAATTGTTGGAACTTCAGGTTCTCAACACT
GTTAAATTTTGAACTTCAGGCCAAAATCACATATTCTCATGGTATGGACATTTTTACAATTTTCTGTACAT
ATGTCTGGACTTCAAGCCCTTACATAATTGTCCATATTTTGAGGAACTTCTGGCATCTCATTTAATTGCT
CATCCATGAGTTTAAGGAACTGCAGGTTCCCTTTTTGAATATAGTGACGATTTACCCAAAATGGTTAATA
TTTATGTATACGTCACTATTCATGTGAATAGTACTATTCATCAAGTCATGAATACGTATCTATTCATCTGCC
AGTACAGTTATCATCCATGTGTACGGCATTATGAACCAATACGGTACTGTTACATCATTAAGGAACTCCA
GGTTCTTATTTACATGTCAGGGATCAAGGACCCTCAAGTCCGATCACATGTTTACAAATACAGTACCGG
AGAGACTGCCAGCTCTCATTTTAATATCATCATCAAGGATCTTCAAGTCCTGATATAAATTGTATGATGA
GGATCAAGGAACTTCTGGTCCTGATCTGCATACTGTAAAAACTCATCATACAGCACAATTAATCCATAA
AATAAATTACTGGTAAATAAATTACTGGTATGGACGATAAACCCGCACCATACTTTAAATAAAAGTAAAT
GTGCAGTAAAGTAAATTCATAAAGTATGAGGGTCAATTCTCATCATACTTTAAGGAAAAGGTAAATTTG
CGATAAAGTAAAGGCAATTATTGGTGGGTTCTTATCAACACCACGATCAAATAATATATATGATGTCATC

TGGAATTGCCTCCCCTTTTTTTTTCTTCGGTGGAATTCCTCTCAAAGCTAGAGGAAATAGGAAACAATA
TAATATTATTACATTAATATATGTAATTAGAAAAGTTGAGACAAACCATAGTGCTCGTCAACCTTCACGT
TTGTCTCCTCCCCTTTTTTTGTCTTCGGTGGAATTCCTCTCAAAGCTAGAGGAAATAGGAAACAATAGA
ATATTATTACATTAATATATGTAATTAGAAAAGTTGAGACAAACCATAGTGCTCGTCAACCTTCACGTTT
GTCTGCTCCATAATTTCGTAAATAAAATTGATGAACATGAACCTGCAGAAGTTCCTCCTGTACACCTCC
ACTTGCTTTTTATGATCAATATAATTGTATAATATTAAAGGTAAAATAGGATAAAGAAAAACAGAGAACT
TATCTCTTTTCCTCAGACCCACTGCTTCCACTTGCCTGACAAAAACTATTCCAACTTATAGATCTGACTT
AGACCTTTACTTCTGCCGACAGTTATGAACTGTTTCATCCAGTTCCTCCATGTCTGAGTGCTGCGCAGA
AGAGAGCAAGTTCCAGTGGGAGAGTTGCGCCCCCAACTCAAATTCCAAGGTGGCCAATGACTCGGCA
GATAGGAGAGGAAGGTAAGAGAGAGTCTCGAACTTCTTCAATCCAATTAGAGACCACTCTCTCTTTCT
CTAAGCTTCAGAAGCCGCGATTCCAAGACCTCGCCGTTTAAGCTCACGGTTCCTTCCAAGCTTCCCTT
CGCGATCCGATTCGCCAGAGCGTCAACGACTTGCCCGACCCGCTCGCGCCGAGAACCGCGAGTATCTC
TCCTTCCCGAGCTTCTCCAGAGATGTCGTTGAGCAGCGTCTTCGTTCTTGTGAACAGGCCATTGACAA
GATTCCCTTGTGCCTCCTTGTTATTGGAAGCAGAGGCCTTGGCAAGCTCAAGAGGTCTCAGCTGGGTT
CGTCGGAATTGCCACCAGTTCCGATCGGTGAAACCAGTTTTTCCGGCTGCTGTCTGAGATTGATCTGCT
GATTGTAGTAGTGGTGGTCCTTGTGGTGGCTGAAGAAGGCGGAGGCCGGAGATGAAAAGCTAGAGCT
GGGTATGGATCCGATTCTGCAAACGGTGCTTCAAGAGGAACTTGAAGAACACGCTGTAGAGCTTAAC
AGCTACGCTTGGTATTTCTCTCTTTTTACCGAAGTCCCATCGTGCTTGTATAAGTTTGGTCTATCTTTCAA
CTTTTCACATCTATCTGCTATGTTTTGTGTGAATATGAATTCGACTCTGTGATAGATATGAAATCAATTTC
ATAGAGAATCCAGTGAAAATTGCTTGCTTGTTGTGTGGCCTGTCAGCTCTGTAGGGAGTAGAATTGGTT
GAGAAATGAGAGGAGGGCACATGAGAGAAGACCCAGGAACCACGTCCGGAATTGGTCTAACTCTTCC
CGCTGGTGTTTGATTTGGGTAGCCAAGTCCTCTGTTAAAAACAGAGAACAAAGCTGAAGATTGTTGTT
GCAGAGAGAAAAGAAGGATTGAAGGTGAGATTTCAGTGGCAGCTGAAGTTTCTGATTTGCAAGAGAG
ATCGAACAAATCTATGAGCAAACTTGGTTCTGCAGGATTTGATCGAATTTCTCAGCTGGTATTTTAGAG
ATATTCGTGCTGATAACGTGTTATAAATCTAGTGAGAAAATGGAGAGGAAATTGAGAGAGAGAGAAGA
GAATGTTTTCTCATTCATTATTGTCATTTCTCTTAGTATTATCAATCTGATATCTATAGGCAAGGAAATGTG
ATGCCCGTGAATGTGCAATAACTTCATGCACCAAATAGTAAGTGGGCTCCACCTTCTTGACTTTTACAA
CAAACATATA

>BGI_novel_G000359, Pp03:2433878-2434575 –
CCGCTTCCTCCATTTCTCTCCTCAATTCTCTGCTCCCAAATTCTCTCCTTAATTCTCTAGATTTATAACAC
GTTATCAGCACGAATTCGCTTTCAACAATTCCAGCTGAATTCTATGATGAAACCCAGCCAAAGAAAAC
ACAAAGAAAAACATAAGCTCTGTTTCTTTCACAGAGAGCATGTGATAGTCTCTCTGCCAAAGTCCACC
AAACCAACATCTCTCAGTAGACGCCAACAAACCAGTTTGGGAGCTACAGCCCATGATCTCGCTCCTGG
CTCTGCTAATCTCACCCTTGCAAACAGCCACCTGCACCTCACAGACATCCAAGAACAACAAGGCTATG
AACTCTCTCTCTCTCTCTCTCTCTCTCTCTCTCTCTCTCTCTATAGATCTGAATAAGAAACATTTTTTTTT
AATTTATTTTAGTTTGCTGGAACAGAAAATGAGAGAGTGCAAACTTCTTTCTTTTGCCATTGTTTTGGT
TCTGTTTCTTGTTAATGGCGTTAATGGTAAAGACCCATATAGGTTCTTCACTTGGAAAATCACTTATGGT
GACATTTATCCGCTAAGAGCAACTCCACCCATAAGGGCAAAGTCTAAGGCAAGGGCAAGCAAGGGCT
GCCACTATTCACATGAATAGTGGCAGCCTTGCCATTTGTGGTTCCACCCATGAGGGCTAAGTCTAGGGC
AAGTCTAAG

>BGI_novel_G000362, Pp03:3215049-3215428 .
TTTAATTTATGAATTTAATTTACCGCACATTTACATTTATTTAAAAGGGTGGTGCGGGTTTATCGTCCACG

CCTTTACTTTTATTTAAATGTATGGAGCAGAATTAATGCCCATACAAGCACCTTAACTTTATGAATTTAAT
TTACCGCACATTTACATTTATTTAAAGTGTGATGCGGGTTTATCGTCCATACCAGTAATTTATTTACCAGC
AATTTATTTTATGGATTAAATGTGCTGTATGATGAGTTTTTACAGTATGCAGATCAGGACCAGAAGTTCC
TTGATCCTCATCATACAATTACATCAGGACTTGAAGATCCTTGATGATGATGTTAATATGAGAGCTGGCA
GTCTCTCCGGTACTATATTTGTAAACA

>BGI_novel_G000425, Pp03:19835059-19837089 -
CCTCTGTGTCAGTGTCCCATATGGATTACAAGGCTAAAGGGGTTTCTAGTTCTATGAGAATTAGGATTA
AAAGTTCTATAGCAATAAGGATACCTCAAGGAGTCTCTTGATGGGCAAGACTCCTTGAGTGATGCATAT
AAATAGAACAGCTCCCTCTAAGAGCCGATACACACAAAATCTCATCAAGTGATTCACCCCATCTGGAA
TCTGAGAATAAGGCATTCGGTGATCTTAGAGAAGAGCTGTCTCCCAGGTGTTTGACAAAAGGCTTCAA
TGGCAGAACAAGGTTAGTGTTCAAGTTATTATAACTTATTAAGTTTACATGTGGTATCAGAGCGTAAGTT
ATTTTATCTTCTACCCAACCAATTTTGTTGCAGCATTTAGAACTTGAAAATTGAAGGATAATTAAAGTTT
TCAAGGTTAAAGAAACTTGTTACAGCAAGAGTTAAAGTTAACACCTTGCTGTGACAAAACAGAAATT
GATCAAAGTTTGTATAGTTCTTGCCGTGCTCTGAGCAAGCCAAAGCAACTCAGACACAACAAGATCTG
TACATTCAAAGTTTTATGATCAAACTTGTGTTGAAAATAAAGGCATAATGAATGATTCACTGTCCAAAG
ATGTGAATGATTTATCGTGCAATTATTTTCAGCAGAACAGCATAAGAGATGAAACATGTAGGCTATCAA
GATTGTTCTTCTGTCCAAAGATGTGGAACTTTTCTTGTACAGGAAGCTTATGTGATTTTCATATCTTAGT
GTTGATGAACATGTTTTGATTTCTTCACCTAAAGGTGAGAATTGATCATGTTTTAAAGATTCTGGGGTTT
TTCAGTTTTAAACACATATATATATATACTATGTGTTTTTCAGTTAATCACAACTCGATTGAAACTCTCAT
TGGCTCCAACTACACCAAATGGAGGGAAGATGTGGAAATTGCTCTAGGATTGCTGGATTATGAGATGG
TCATTGAAGAAGAAGCTCCTGCAGAACCTGCTGCTACTGCATCTGCTGAAGCAAAAGCAAAATATGCA
AAATGGATTAAAGCCAACAAGATGGCTATTCTAATCATGAGGAGATCAATTTCACCATCTGTCAGAGGA
AGCATCACACCATCTGATAATGCCAAGAAGTTCATTGATTCTATTGCGGAAAAATTTCAGGAATCCAAG
AAAGCTGAAATTGGTACTCTGATGGCACAACTTACTGATGCAAAATACAATGGGGAGAAGTGTGTAAG
AACACACATATTGAATATGCTGGAAATTGGGAACAAGCTGCAAGCACTCAAAGTCAATGTGGATGAAA
ATATGATGGTACACTTTGCAATAAATTCTCTACCAAGCACTTTCAAGCACCTTAGAAGCACCTATGTAG
CACAGAAGGAAATATGGACAGTAACTGACCTCATAGGCATCTGTGTGCAGGAAGAACAAAATATGAA
GAAAGATAAGGCTGAAGAAAGAGTTAACATGGTTCACAACTTCAAACCCAAAAAGGATTTTGGAAAG
GGTAAAATTGCTTGGAAGAAAAACAAGGAAGAAAAGGGAAGTAAGGGGCTAGAACCAGAAGGTTTC
AAGTGTTACTTCTGCAAGAAATTTGGTCATATGAAGAGGAATTGTGACAGATACAAACGCTGGCTAGA
TAAACAGAAGGCTAAAGGGGTTCAAAACAAAGAGGCTGCCAAGCAAGGCTGAGATGAAGGTGTTCG
TGGGAAATGGAGAAAGAGTCAAGGTGGAGTACATTGGATTAGCTAAGATCGTTTTGGAGTCTGGTTTT
ATTTTGGAGTTAGTAGATGTAGTTTATATTCCTTTTATGAAAAGGAATCTTATTTCAGTTAGTAAGCGTGT
AAAATCAAACTTACAGTTTGAGTTTGATGAGTCTGGATTCTCCATTTTCAGAAATAAAGTTATGATTGG
TA

>BGI_novel_G000503, Pp04:17638321-17641467 +
AGAAAAGAAAGAAAGGGGAGAAGAGAAAAAGAAGAGAGAAGAAGAAGAGAAAAGAAGAAAGAA
AAGAAGAGAAAAGAAGAAAGAAAAGAGAGAAGAAGAAAGAGAGAGAAAATTCAGTGAGCCTCACA
TATTGTAAACTCTAAAGTTGTAGCCCTATTATTTTATATAGTGAAAAAGTTACTGCTGCTGCTCTCCGAG
GACGTATGCATAGCCGAACCTCGTTAAATGCTGTGTCTCATCTACTTACGTGCAGCTCAATATTCATACA
TATTCCAGGTTATTTTTATAACACGTTATCAGCACGAGAAGCTCTCAGGTATAATTTTTGTGCTGCACTAT
TATCTATACTACTAACCACTAACAAAAATGGACCCTTGGTAATACTAAACATATTATCAGTGGGAGACCG

TTACTAATACTAACTTTTTCTTATTTTATTCGGTGGTGATTTTAACCCCACATTTATTTACTGTATGGTGTA
GGTTTATTACCCATACATGCACCTTTACTTTATCACAAATTTATTTTACCGCATATTTATTTTATTTACTGTA
TGGTGTAGGTTTATTACCCATACAACAGTATTTATTTAAAAGGGTGGTGCGGGTTTATCGTCCACGCCTT
TGCTTTTATTTAAATGTATGGAGCAGAATTAATGCCCATACAAGCACCTTAACTTTATGAATTTAATTTAC
CGCACATTTACATTTATTTAAAGTGTGGTGCGGGTTTATCGTCCATACCAGTAATTTATTTATCAGCAATT
TATTTTATGGATTAATTGTGCTGTATGATGAGTTTTTCCAGTATGCAGATCAGGACCAGAAGTTCCTTGA
TCCTCATCATACAATTACATCAGGACTTGAAGATCCTTGATGATGATATTAAAATGAGAGCTGGCAGTCT
CACCGGTACTGTATTTGTAAACATGTGATTGGACTTAAAGGTCCTTGATCCCTGACATGTAAATAAGGA
CCTAGAGTTCCTTAATGATGTAACAGTACCGTATTGGTTCATAGTGCCGTACATATTGATGATAACTGTA
CTGGCAGATGAATAGATACGTATTCATGACTTGATGAATAGTACTATTCACATGAATAGTGACGTATGCA
TAAATATTAACCATTTTGGGTAAACCGTCACTATATACAAAAGGGAACCTGCAGTTCCTTAAACTCATG
GATGAGCAATTAAATGAGATGCCAGAAGTTCCTCAAAATATGGACAATTATGTAAGGGCTTGAAGTCCC
GAAATATGTACAGAAAATTGTAAAAATGTCCATACCATGAGAATATGTGGCTTTGGCCTGAAGTTCCAA
ATTTAACAGTGTTGAGAACCTGAAGTTCCAACAATTAAATGGACAACCCTTGAGGTATTATTGCAAATT
GTGGTATGCCACATATTTCTTTGGCATAAGAAGATGATGAATTTAATAAAGTTCGATTTTGTTATAATCGA
CACCTCAAGGAAGAAATATATTTTGTGAATTCCGGTTGTTAAGAATACACCGCGAAATGGATAATATTA
ATATAAACCTCAAATAATGAATTGAGACTCCCCACATATTTTGTTATATCTGGGCCGGAAGCCCATGGAT
CCAAATATATGAGAGATCCTAAACTTGAAGTTGTGGGTATATAGTAGTTAATGCTGTTCACTACTATATTG
CGATATTATCATGGCTTTTACTCAATTCATATTTCATGTCCATTTGAAAAGGGCAAATAAATTCTGAGTTT
GTGCCCAGGCCATAAGTTCCGGGCTACCATACGTTGAAATATGAAATTTGGGAGAGTCGATGTGGTTAT
TTGAACCTATAAGGCACAAGGTTCCAAATCGTCATTTTGAAAAGACGTAAAAACCACAGGTGCAAGTT
TAAGAATATGCGCAATTATTATAACAGGAACATATAACAGATAGATTTTTCCACCTGCAATGAAGGTGCA
GGCCCTGTGAAATCTATAAAAAATTACATTATGTTCTGGAAGCCTCTGCAGGAAGATGACGGCGCCTCT
TAAGCTTAGCCATGAGCTTCTCGTGGTCTCCCAAAATTCTTTCATTGGAGACCTGCAGCATGTCTAGCC
TTCTCAGTGTATCAACAGAGTACGAACTCACCAGTTTCAACAAGGCATTATTCTCTCCTTTAAGTTTCT
CAACCTCCTGTTGAGACTCATGAAGCATTCTTTGGAGAGACGAATTTTCAGTTGTTAAATTCTCAACTT
CGTTTGCTCTGGCACGCAAACGATCAGCCATGTTAGAAACAGAAGCAGCACTCTGAATGCTAAAAGC
CATTGAGTCATCAATAGCCTCTTCCTCTGATCTCCCTGTCAACAACATTTCATCCATTGGAGTAATGAAA
TTCCTAGCTACTATGACAGCAGTAGCATCATTCATCATCACAGAATCATTAACTGTGAGATGACGATTTT
TGGATACAAAGGATGGACGCCAAACTTGGGCATCACTGGTTGTTGTGGGAGCATCATTTAAATTGAAA
TAATTTGGGCAGGAAGAAGAGGAAGCCATTTTTTTAAGAAGGTTTCGAAGAAAGTCTTAGAAAAACT
AAAGGTTCGGAAAATGAAGGAAATTGAGTTGAAACGCAGTTTGCAAAGGCAATATCTATAGACGAAA
ATGTGGCAACTTTTCAGGACCCCAATGTCTTCTCGAATCCCCATATTATCTTTTTCTTTCCCAGAGTCCA
AAACTTCACGTGGCCTCTAATAATGCCTGTCGGCACTTTCGGCGTTTTTCAAAGTATTATTTTCAAAGC
CGATCTTGCTTTACGGATTTCACGGAACTACTTTTTCAAAAGTATCCCGAGAATCTCGCAATTTTCCTAT
GGTTAATTTGAAATTCACCGGTTCTACCTATTCATTGTATTTGTGTATAAATGTGTTACTAACGAAATTTT
CTTTGCAAACGACATCCAGTTTTCTCCATACCTAGTGATGCGATATCTACTTGTTTTTCTTATCAGTGAA
CACTTAATATGCCTGAGAAGCAAGACTATCTCTGATCATCCATTCAGGAAGCAAGACTATCCCTGATCA
CGTTTCCACTACATCAGGAAACTGTGAAGGCGATCTTATGCTCTAATCTCCGGAAGTCCTTCTAGACTA
GGAGATATGAGAGCTTATTGTCTG

>BGI_novel_G000659, Pp06:13132808-13133357 .

GCTAGGAACCACCAAGGAATCTCTGGTTCTTTGAGTTCCAAGAATTCCTTTCCTTTCCTTTCCAATGAA
ATTTTCCATCAAATACAAGACATGGGACAAAGAGGGAATTGCTTATCTTTGTGAGCTTTGAATTCTCAA

ACTTGGATTGTCACCTCCCTATTGTCTTTGCTTGTGGTTTTCCTTTGAATATGGTCCAAAGAAAGGTACC
AAGCAAGCTTGGAATCCAAGCTGATCATGTTAAATTTGAGAAGCGGTTGGCAAACCTGAAAACATCTT
CTCAGTTCCAGGATGGCAAACATAGGGGAGCTGATTTGAAGGAAAAAAAAAAATGAAGAAATCTAGAT
CAATCAAGCTTTCAGATATTGAGAGCTTGAGATCATCACCTTTGAGAAAGAACATATCCCAACCAGGA
AAGCCACCACCACCATCTCTCAATGTTCCAAACACTGCAGCTTTTCCTCAGAAGCAGCCTATGAACAA
AACAACTTATGGCTCACCAAACTATATGAAGCCCACCAGCTGTTCTGATGCAAGGAAGGAGCAATCCC
AGA

>BGI_novel_G000750, Pp07:1984294-1989082 +
GCTTTTCAAACGTGATCAATCAAAGAAAAACATACATCAAGGATATATTATTTTAGTGAATGAGAATTC
ATCAATAATATCTAGAAATTCATACACTCAAACATCAAAATAATAGAGTATTCATGTTTAGGGCTTCAAC
CTAAGCCCTAGCTATGGAGTTTAGTTCCTCTACTTATTTCAAACACAAATTCGAAATAAAACTAGAAAG
GGTTTACAGATTTTGTGAAAGTGATGGAAGCTCAAGGTCTTCAATGGTGGAAACCTTCTTCTCCTTCTT
GCTTGGCTGCCCTTGTCTTCCTCCTCTAGCTCTCTAGCTCCAAAATCTCCTCCAATCTCTGCAAAATTGA
ATAATCCGTTCAAATGTGGTTGATGCCTTCTCTATGCTCCCCCTCTCTCTCCTCCTTTCCAATTCACCCAT
CAATATTAAACTCATTGTTGTAAAAGTGGAGTCCCCAAAATGCCCCCCTAAATGCCTATAAATAGGGCT
TCCCTTACTTGTAAGAACACACATTCAGAACACATATTAGAATGTGAAATCAGTTTCCATTCCCTTCTCT
CTCTCCCAATTTCCTCTCCATAATCTCTTAGTTTTACAACACGTTATCAGCACGAGTCTTTAACCGGCTG
AGTTCTGTGATGAAACCCATTTAGATAAACCAACAGCAGCAAGCAACAAATATCAAAATCACATCACC
AGCTCTGATCAGTATTTCAGAATCTCTGATTTCTTGATCCCAGAAATTGATCAAGACACCCAGTTCGAA
ACCAAGCCCCAGATTGCTCACCCAAAACCAACCCAGTTTTGTACAGAGCCACACGGATTGCTTGCCA
GTGTCCTGCCATGCCACCACAGTGAGGCCCTCGACAGCAAAAGCGCCAAGCGCACCCAACAAAGAG
ACCAAAGGGCATGATCAATTCATAGTTCCTGGAGATAGAAAATATGATATCTGAAAAAAAAAAAAAAA
AAAAAAGAACCAGACAGTTTCATCTTTGGGTAAAATCGATTCTTGGCTAACTGGCATGGCTATGCTTCT
TAAGGCTTGCGTGGCTTCTCTCACACAAGCTTCTACTGCTCACCATAAGCACTTCCTGGCTCCATCTGC
ATTCAACTTCAGCACATCAGGAGCGGCAGGAGACCTTTGAGCTGGCTGTTGCTGAGGTCCAGGGCCG
TATTGAAGGCAACGTCGACTAGCCATCCAAGGGCTTAAGCCTCATCGCATTATCACACTTCTCACCAGC
AAAAGAGAAGCAAAAGCCAAAACGAAACAGAGGAAGCAAAATCCACTTTTTTTGTCTGATCGATCAG
CCATCAATGGCCGCGAAGAACTTCAAGCACGTCATCCTCGGCGGTGGAGTCTCAGCTAGATATGCAGC
TCGGGAGTTTGCCAAACAAGGGCTTATCTCTTCCCCGAGTCTCCTGCTAGACTTCCTGGGTTTCATGTT
TGTGTTGGAAGTGGAGGAGAGAGATTGCTTCCCGAGTGGTACAAAGAGAAAGTTTGGCAGGGTCTGG
TGTCCAGAACATGAGTCAAACGACGTCGTCGGTTGGGAGCTCGAAGATACTAAGGCTGTTTGGGGTG
ATCTTGGAGTGCCAGCAGCAGGCTGACCACGAGTCCCAGCCATCCATCCACTCCCGATGGCTCGTCGT
CTTTGTCCATGTCATCGAGCCAGGGTCCAACCCCACACCTGACTCGGGTTGCAGCGCGTACACGAGAT
TTGTGAATTTGGACCGGTGGCTCGCCCTCCAGCTCCTCCCCACTGTCAAGATTGGTTTGCTTCAGCTTC
TGGAGGAGATTCTCCTCCATTATTTTGCATACCCATCTTTTCTTGTGAGTGGAGACTGCAACAAGCTTT
ACCAATTTGTTAAGGAACAAACAGAGATGCTGACTTAAGTCTAATGGCGGGCCACGCGATAAACACAA
GTTGGCTGGACTGCACATGTTGTCGAGTGCGCATGATTACAGCCCCACCCACCGAAACATTAAAAAAA
ATAATGGCATAAGCGAAAGAATAAGCGATAAGATCTCTGCTCCCTTATCTTCTCTATTATTTCATATTATAT
TTATCTGCTTTACTTTTACTAACCATTAACAAAAATGGACCCTTGGTAATACTAAACATATTATCAGTGG
GAGACCATTACTAATATTAACATTTTTCTTATTTTATTCGGTGGTGGTTTTAACCCCACATTTATTTTATTT
ACTGTATGGTGTAGGTTTATTACCCATATAACAGTATTTATTTAAAAGGGTGGTGCGGGTTTATCGTCCA
CGCCTTTACTTTTATTTAAATGTATGGAGCAGAATTAGTGCTCATACAAGCACGTTTACTTTACCGCAAA
TTTACTTTTTCTTAAAGTATGACGAGGAATTGAACCTCATACTTTATGAAATTTACTTTATGAAATTTACT

TTACCGCACATTTATTTTATTTACTGTATGGTGTAGGTTTATTACCCATACAACAGTATTTATTTAAAAGG
GTGGTGCGGGTTTATCGTCCACGCCTTTACTTTCATTTAAATGTATGGAGCAGAATTAGTGCCAATACAA
GCACAATTTACTTTACCGCACATTTAATTTTATTTAAGGTATGGTGCGGGATTAACGTCCATACAGCAAG
CAATTTAATTTACCGCACATTTACATTTATTTAAAAGGGTGGTGCGGGTTTATCGTCCACGCCTTTACTT
TTATTTAAATGTATGGTGCGGGTTTATCGTCCATACCAGTAATTTATTTACCAGCAATTTATTTTATGGATT
AAATGTGCTGTATGATGAGTTTTTACAGTAAGCAGATCAGGACCAGAAGTTCCTTGATCCTCATCATAC
AATTACATCAGGACTTGAAGATCCTTGATGATGATATTAATATGAGAGCTGGCAGTCTCTCCGGTACTGT
ATTTGTAAACATGTGATCGGACTTAAGGGTCCTTGATCCCTGAGATGTAAATAAGGACCTAGAGTTCCT
TAATGATGTAACAGTACCGTATTGGTTCATAGTGCCGTACACATTGATGATAACTGTACTGGCAGATGAA
TAGATACGTATTCATGACTTGATGAATAGTACTATTCACATGAATAGTGACGTATGCATAAATATGAACCA
TTTTGGGTAAACCGTCACTATATACAAAAGGGAACCTGCAGTTCCTTAAACTCATGGATGAGCAATTAA
ATGAGATGCCAGAAGTTCCTCCAAATATGGACAATTATGTAAGGGCTTGAAGTCCCGAAATATGTACAG
AAAATTAAAAAAATGTCCATACCATGAGAATATGTGGCTTTGGCCTGAAGTTCAAAATTTAACAGTGTT
GAGAACCTGAAGTTCCAACAATTAAATGGACAACCCTTGAGGTATTATTGCAAATTGTGGTACGCCAC
ATATCTCTTTGGCATAAGAGAATGATGAATTGAGGCTCCCCACATATTTTGTTATATTTGGGCCGGAAGC
TCATGAACCCAAATATATGAGATATTAGCGTGACTTTTACTCAATTCATATTTCATGTCCATTTTGAAAAG
GGCAAATAAATTCTGAGTTTGTGCCTTGGCCAAAAGTTCCGGGCTACCATGCAATTTGGGAGAATCAA
TGTGGTTATTTGAACCTATAAGGCACAAGGTTCCAAACCGTCATTTTGAAAAGACGTAAAAAACCACA
GGTGCAAATTTTAAGAATGTGCGCAATTATTATAACAGGAAAGGAGCAAGTTGAAACGCAGTTGCTCC
AATCAAGTTTTGCAAAGGCCATATCTATAGGAGGAAATATGCCTACAGTTTCCAGGATCCCAATATTATC
TCAGATCCCCATATTATCTTTTTCTTTCCTAGAGTCCAAAACTTCACATGGCCTCTGATAATGCATGTCG
GCACTTTCGGCGTTTTCAAGTATTATTTTTGTCAAAAGCCGATCTTGCTTTGTGGATTTCACGGAGCTAC
TTTTTCAAAAGTATCCCGAGAATCTCGCAATTTTCCTATGGTTAATTTGAAATTCACTGGTTCTACCTATT
CATTGTATTTGTGTATAAATGTGTTACTAACGAAATTTTCTTTGCAGAAGACATCCAGTTCTCTCCATAC
CTAGTGATGCGATATCTACTTGTTTTTCTTATCAGTGAGCACTTAATATGCCCGAGAAGCAAGACTATCT
CTGATCATCCATTCAGGAAGCGAGACTATCCCTGATCACGTTTCCGCCACATCAGGGAACTGTGAAGG
CGACCTTATGCTCTAATCTCCGGAAGTTCTTCTAGACTAGGATAAATGAGAGCTGGTTGTTTGCTCCCA
CCAGCTTCCTTCCTTGATTGCTGAGCTTGTTGTCTGCTCCCACCAGCTTCCTTCCTTGATTGCTTACCTT
CTCTTGCAATCAATATCACATTATTTTTGCATTATTTCTCTTTTTATAACTCTCTGTTCATAAGGGATTTTA
TTTCTTTAGAATGAACATCTGAAGAATGAATCCATGCAGTGATCCTAACTCTGAGAGTTATTTGTTTTTG
ACAGAGAAAAGAATTGTGATTTTTGCTCTTGCAGGATATAACAAGATCATCAAGCGATACTTTATATTTA
CTGGGCCGATACGAGCTTGAATGATACTTGAGAAAAGTTTCTCCCACCTAAGGATGTAAGCAATACTTG
TGTTATTTTATGCTTATATACCTATTTGATATTTTATCTTGAAAGGTAACCAAATGGGAAGATAAGTCCGT
TCCAAAAGAATGGCTTGTATGTATCCATGAATTATTAATGCAAATTTTACAGATTGCAAGTTGGATACAT
TGATA

>BGI_novel_G000830, Pp07:19265483-19265795 +
TTTCATGGTTCTCTTCCTTCCTGGCTCTGCTAGTCTCACCCGCACAAGCAGACACCTGCACCTCACAGG
TATTCATTAACAGCAATCTCTACTCCAGCTGCACCGACCTCCCCGTCCTCAGCTCCTACCTCCACTGGA
CCTACGATACCTCCAACTCCTCTCTGTCCATCGCCTTCATCTCTACACCCCCCCAATCTGACGGCTGGG
TAGCGTGGGGCATCAACCCCACCTCCACCAAGATGGCTGGGGCACAGATACTCTTGGCCTACAACACA
GATAGTGGGATCCCAACCGTCAAAACCTTCAACATCAG

BGI_novel_G000853, Pp08:3842056-3842798 .

TACAGCAAGCAATTTAATTTATGAATTTAATTTACCGCACATTTAATTTTATTTAAGGTATGGTGCGGGAT
TAACGTCCATACAGCAAGCAATTTAATTTATGAATTTATTTTACCGCACATTTACATTTATTTAAAGTATG
GTGCGGGTTTATCGTCCATACCAGTAATTTATTTACCAGCAATTTATTTTATGGATTAATTGTGCTGTATG
ATGAGTTTTTACAGTATGCAGATCAAGACCAGAAGTTCCTTGATCCTCATCATACAATTACATCAGGAC
TTGAAGATCCTTGATGATGATATTAATATGAGAGCTGGCAGTCTCTCCGGTACTGTATTTGTAAACATGT
GATTGGACTTAAGGGTCCTTGATCCCTGACATGTAAATAAGGACCTAGAGTTCCTTAATGATGTAACAG
TACCATATTGGTTCATAGTGCCGTACACATGGATGATAACTGTACTGGCAGATGTACTGTACACATGAAT
AGATACGTATTCATGACTTGATGAATAGTACTATTCACATGAATAGTGACGTATGCATAAATATTAACCAT
TTTGGGTAAACCGTCACTATATACAAAAGGGAACCTGCAGTTCCTTAAACTCATGGATGAGCAATTAAA
TGAGATGCCAGAAGTTCCTCAAGATATGGACAATTATGTCAGGGCTTGAAGTCCCGAAATATGTACAA
AAAATTGTAAAAATGTCCATACCATGAGAATATGTGGCTTTGGC

>BGI_novel_G000906, Pp08:14722502-14725198 +
GGAGCCCACAAGTTAGAATAGGTTTTGCCTATAAAAGGAGTGATCCCTCTCCATTGTAACACAGACAG
AAACATTCAATAAGATAACAAAACATTCTCTTCATTCTCTCTCTCAATTTCGCTCTACATTTATAACACG
TTATCAGCACGAATTCGCTAAAACAATACCAGCTGAATTCTCTGATGAAAACCAAAGTTTCTCCCTTGA
ATTTCCTTTCTGGGTTCTCTGTTTTCTATATGCAAAAGCAGCAACACCTAAAACCAGAAAAAGAAAGA
CGAAAACCTCTCTCTCTGCCACCCCTTGAAATTCACAGATATCTGTTTTATCAAACATCAAAGACCTTT
TGGTCCCAAGTCAATATCATCAACCTCAGATCCAATTGAATCAAAAAAAAAAAAAAAAAAAAAAAAA
AAAACCAACCTTTTCTGTGTTCCTGTGGACTGTTTTTCTCTGCTCTGGATTGCTTGGTCTGCCCAGTTT
GGGTATTGTTAGAACAACAAGATCAGTGGCAAACCCATGTCCGGAAATTCAGTTCAACTCATGCTTCA
GCTGTTCTCAGATATGGAACATCTCTTAGAACAGCGACGTCGTCAACCCAGCGCAGCGCAGGCCCGCA
TCGCCGAGAGGCCGGCGCATGCGCCGTCATGGCCCGTGAGAGCGTCCCCGCCGACATCCAGGTGCTG
CCCCACTTGCACGTCCACGCCCACGCACACCTCGTCCACGTCCTGCTGGTTCACCAATCCAGCGAAAA
GGTTTGGAAGAGAGGCCAACTTCTTGGTGGTAAACACTTTGGTGCCACCAGTCGCTCAACAAAAGGA
TCAGCTCACCGTCGTAGGACCTCATCAGTCTCCCACAAGCCCACGGCTCGAACCAGGAAAGCCGACC
CAACCAACAACAGCGGCGGCTGCAGACAACAACGGCGGCGCTGTCCACATCATCAGCAACTGCAGG
CATGGTGATGGTGGTGGTGGCTACAGTGACAACACTGTCTCCTCAGATTTCTTTTATTTGTTACAAGTA
AGCTCTGAAGCCTAAGCCCAAGGAAGAGAGGGAGCTGACGCACCTGTACGAGCGTCGACAGTTCCCC
TACAAGGGCGACGAAGAAGCCATGGAAAGAAGTCAGTGTTCTTGCGCTGCTATGCTCTTTACCTGGCC
GGAGAAAAGCGGAAAGAGGAAGAAATGATAGAACTTGAGGGGCCCCTAGGTAAGAGCGACGTTGTG
AATGGTGAACTGGTTTCTCTAGAGAGGGAGTTATCAACCCTTCGCAAGAATGGGACCGTTGACCCTTT
TGGACTAGGAGAAATGGTGATGGTGACGATGCAGAGGTCGGATCCGTGGGTGACGGCGTAGTTGACG
TCGGCGATGATCTTGGTGAGGGGGAGGAAGGCTATGGAAGACAAGGTTATGGTTCATGAGGCTAAGG
ATGCTCGGTTGCTTGAGTTGTATTGAGTGGTCAATGGCTGTGGTGGAGAGAATATGCTGTCGGTACGCA
TGATCACAGCCCCCACCCACGATATACTGTCGGGCACGCATGATCACAGTTGCTCAGCAACACAGGCA
CCGAATATGCTGTCGGCTATCATGGGTTGTGCTATCCTGCTGCACATGTAAAACCACCCACCGAAAATA
AAGGGATAAGTACTCTGACTCACTCTATCTCCTTCAATTAAATACATTATTAAAATACCTACAGAATTTA
ATACTCATGATCAGTGTAGATTGCAGGTGGAAAGAAGAGAAGCCAACGTGATATATCTAGCTTTCACTA
ATATATCAGTATAATAATATTATACTATTTGATCGTGGTGTTGATAAGAACCCACCAATAATTGCCTTTACT
TTATCGCAAATTTACTTTTACTTAAAGTATGATGAGGAATTAACCCTCATACTTTATGAATTTAATTTACC
GCACATTTACATTTATTTAAAGTGTGGTGCGGGTTTATCGTCCATACCAGTAATTTATTTACCAGCAATTT
ATTTTATGGACTAAATGTGCTGTATGATGAGTTTTTACAATATGCAGATCAGGACCAGAAGTTCCTTGAT
CCTCATCATACAATTATATCAGGACTTGAAGATCCTTGATGATGATGTTAATATGAGAGCTGGCAGTCTC

TCCGGTACTATATTTGTAAACATGTGATTGGACTTGAGGGTCCTTGATCCCTGACATGTAAATAAGGACC
TGAGGTTCCTTAATGATGTAACAGTACCGTATTGGCTCATAATGCCGTACACATGGATGATAACTGTACT
GGCAAATGTACTGTACATATGAATAGTGCCGTATACATGAATAGTGATGTATACATAAATATTTACCATTT
TGGGTAAACTATCACCATTCAAAAGGGAACCTGCAGTTCCTTAAACTCATGGATGAGCAATTAAATGA
GATGCCAGAAGTTCCTCAAAATATGGACAATTATGTAAGGGCTTGAAGTCCAGAAATATGTACAGAAA
ATTGTAAAAATGTCCATACCATGAGAATATGTGATTTTGGCCCGAAGTTCAAAATCTAAGGGGATTGAA
TGTTCATACCATGAGAATATGTGATTTTGGCCAGAAGTTCAAAATCTAATAGTGTTGAGAACCTGAAGT
TCCAACAATTAAATGGACAATCCTTGAGGTATTATTGCAAATTGTGGTACGCCACATATTTCTTTGGCAT
AAGAGAATGATGAATTGAGG

> BGI_novel_G000473, Pp04:12563396-12565812 -
TCTATTTTCACGCTCTCTCTTCCTCCTTCCCTCACATCACGTTGCTTCTTCTCACAATCTCTTCACAGAG
CCCACTCCCCTCCTTCTCTTCAATTCTGCCTACAGTAGTTTTTTCTTGTTGGATCAATTGAAGCGCATTG
CTGAGCCACAAGACTCAGAAAGGGTATACCTACCCAAAATGTGATGGCTGCATGCAATTTATATTTGCA
TGTGCCGGATGGGAAGGCACGGCCCATGACACAAGGATATTTCTATCGGCTTTACGTGATCAAAGTTT
GAATTTTCCTAAACTTCCAAATGGAAAATTTTATCTAGTAGATGCGGGATACCCACAAATGAGAGGCTA
CTTAGGACCATATAAAGGTGAAAGATATCATCATCTTCCTGATTTTCGTAGGGGTGTTCAACCAATGGG
TCATAAAGAGGTATTCAATCATACACACTCTTCTCTGAGAAGTGTTATTGAACGCACTTTTGGAGTATG
GAAAAAGAGATGGAGTATTTTACGCGACATGCCTAGTTATCCTTTTGCAAAACAAGTCAAAATAGTTAT
TGCAACCATGACACTTCATAACTATATAAGAAGACATGCCAAACGTGATTGTCATTTTGATGAAAGTGA
AGATGACCTAAATGGTACTCAAGATGAAGAGATTGAGAGAGAGGTTGATGCACAACAAGAAGATGGT
CCTGTAACACAAGAAATGGAGGCATTGAGAAATCATATAACCGCAAGTTTAATGAGTTCATCTGGTTAG
AAATGTATATGATTGCTTATTTATGACTAGTTTAATTTTTGTTATGACCATATTATTGACAATATATTTATTT
TTAATTGAATACTTTATTTATTTTATAATTAAAA

>BGI_novel_G000725, Pp06:27354244-27359524 +
ATGGAATTCACATGCTTGTAAGGTGTCATCCGCTCCAAACAAGATCAAATTCAATGCATATTATCTAAAA
AGATGATGAAAGCCACAAGAAAAGGAAGAAAAAGAGAAAACAATTCAACAATGGAATTATGGGCAA
CCACACCAACATAGACAAGATGAAGTGGGAGTTATGGATTGTATTAAAATAGGAATGGTGGTGTTGGA
ATTCATCATAATGTTCCTCCCTCTTAGCCCTATAAACAGAGAAGTCTTCAAGCTTCAAAACACACACCA
AGCTCACAATCTTCTCTTGAGCTCTCTCTCCTTCCTCCAATTCTTCTAGTTCTAAATTTCTGTTTTAGTTT
TATTTTTTGGGGAACTAACTCCCTAGCTAAGGCTAAGGGGGAAGCTTTTAATATGAGTATGTAATTTTTA
TTAATTTGATCCAAACTTAAGCTTTAATGTGAATTTGTAGTTTTGTTCAAATGGAATTTGTAGTTTTTCTT
TTTAATTAATTGATGAATGTTTGATAATCTATTTCATGGGTTCAATAATGTATTTCCCATTGTGTAGTAAAA
ACTTTCATGCTTTTAAAAGATGAGACAATTACTAAGATAGATAGAATTTCGATGAAGATTATTTTTTGAT
AGTTTGTTGCCAATGGTACAAGTTATGCTTAGAATACTTCTAAAGGAAATTAAAAATAGTTTTAATTATT
TTGAACAACTACAATGGAATGGATCAAATTAATAAGAGTTGCATGCTTATGTTAAAAGCGGAACCCCA
AAGCCTTAATGTTTTAATCAATTTAATTTTCTTTAAATCAAATCTTGCATAAATCTACTTCTCAATCTTAAT
TGTTCTATCTCAACTCTGTTATTTTCTTTACACTACTCTCTGTGGATACCACACTTGGACTTCCAAACTA
CACTGCTATACGACACCTTCACTTGGGTTTTGCAAAGTGGTACAAATCAATTGGCGCCGTTGTTCGGG
AGCGGCTTGTCAAAGAGTACAGAGATCCCCTTGGTTGGAGGTTTGCTGCTTATGTGAAATTCTTCACAT
CCGGAAATGAAAGGAGCTTATGAGGAGCGTTGTGCTGCTATTGGAAACTTCAAACAATAGTATTCTCC
TTTTTCCAACACTTATAATCCAGCAACAAGGAATCATCCAAACTTTTGTTGGAGCAATGAGTCACAACA
TCAGAAGCAAAATCAAAGGTATGATACTTTGCTTGGTCAATTGGGTGAAGAGAATAGGGAAATTGGCA

GCTTTACCCCTGGCTCACCTAATTTATTCACTAATATCCTTATTTTGAAACTCAATCACAAATGCCCCTTA
TAAAAACTCCTTATTGACAGCCTATTGCCCATCCTTCACTTCGTTTTTTTTCCTTAGTTGTTGCTCTTCCT
CTTCCCCTTCCTTTTCTTCCTTTTCTTCCTTCTGCCAAACCTCACAAAATCCACTTCAACCATAGAATCT
CCCTAAACTTTATCTCATCTTAGTTATATCTTCCAACTAGGAGCTGATTTCAACCTCAAAGTTCAACCCC
AAATTTGGACCGGACCCTAGCCAAGGCGCGACTCTCCACCTCCGTTGTCGGCCTCCACAACAAAGCC
ATAGTCTTGACGTGCCACCTCCATTGTCGACTTCCACCACAAAGCCACGGCACCTTCCTTGTCGATCTT
CACTGTGAAATGGACACAATTGCAATTTTGGTTTGCTACAATGGCAAATGGGTAACCTCAAAGAAGAT
GTGCACATATGAAGGGGGTGACTCAAAAGGCATAATAGTTTCGCAAAACATCACGTTTGGTGAGCTTT
TGGAACGGGTGCATAAGATTGTTAATAGAGACAACAGGGAAGACAAGCTTTGTTTAAAGTTCTCAATT
TCAATATCCTCGAATGAGTGTAAGCATATCAAGATTGAGGACAATGATGATGTTTAATTTTTTTATTAAG
TACATTTGTGAAGTAATTCCTTCAAAAGTGGCTTCTTTACTGGTGAGCATAGAAGACAGAGGAGTGAC
AAATGTTGTAGGTGATCGTATACATATCACGACGGATATGAGTTGGATGGCCTGTTCTTCAAATGCCATA
GTTGAAAGCAATGGTGATGTACATGGTGATTTTGGAACAGAATATTTAGATCTGATTGATTTTAAAAGG
GTGGAGGGGATGCATAGTGGTGATAATGGTACGGAAATGAATGGCTTGCAAATGCACTCAGCCCATCC
TATTTTGGCCCATTTGGAGACATTTTCACAAGTGGGTGTGAGGGGTTTTGGAATAACGTCTGAACTTTC
TATTCGACATAATTAGAGGCAAATGGGTGAACAAAACGTTGACAATTTGGGTATTGTAAATGATGAAGA
AGAAGATATTGAAAGTGCGCATTCACGGAATAATTGGGATCCAAAATTGGAAGTTGGGAAAATTTTCT
CTAGTAAGGAAGCCCTGTCCAACAAGTTACAGTTGGTGGCTGTGAGAGGTCACTTTGAATTTAAGGTG
AAACAGTCTTGCAAGAGTTGATTAGTTGTGGTTTGTTCTCAAGGCCCATGCCCATGACGGCTTCGTGC
ATCTAGTTATGGAGAAAAGAACTTCACGATTGTGAAATATAATCCAGTTCATGAATGTGATATGAGTTTT
ATACATGACAAGCATCGTCACGCAAGTGCTAAACTCGTTGGTAATGCAGTGAAGCAGAAGTTCAAAG
ATTCTCGCACAATATACACAGCAAGTGATATAATCAAAGATGTGAAACAAAACTTTGGTGTCCCCATTA
ATTATTGCAAAGCTTGGAGATCGAGGGAGTTAGCTCTAATATCCATTAGAGGTTCAGTAGAGGAGGCAT
ATTCTCTCCTTCCAGCTTATTGTCATGAATTAGAGCGTGTGAATTCCAGCACAAGGACATACATCCACA
CCGATGAGAACAATCACTTTTTGTATTTTTTATGGTTGTTGGGGCATGTATTAGAGGGTTTCAATCTTTG
AAGCGGCTAGTCATTGCTGTGGATGCCACGCATTTAAAATGTAAATATAAGGGTGTCCTTTTTGTTGCC
AATGCATTTGACGGAAATCGAAATATATATCCTCTTGCTTTTGGAATTGGGGATTTAGAGACGGATGCAT
CATGGCATTGGTTTTTCAGTAAGCTTCATGAAGTCATTGGTGAGTGTCCAAATCTTGTGATTATTTCTAA
TCGGAATATTAGCATCGAGAATGTGTGGCATAACATTTTTCCAAGGCGCAACATGGCATATGCTTTTACC
ATATGAAGAGGAACATGAAACGCACTTTCAAATTGAAAAAAACGTGATAAATTATTGCTACACTTTGA
ACAAGTTTCAAAATCTTATGGCATCGCTGAATTTGATCGTCATTTTCGCAAGATCAAGGGAAATGATGA
GGTTGCTCAATATCTTGAAAGTGCAGGATTACACAAGTGGTCTAGAGCTCATAGGG

>BGI_novel_G000833, Pp07:20699156-20699568 -
ATATTTTCCAAGTTGAGAAATAGAGTGTTATGAAATGATGAAAGAAAGCATGCTGCATTTGAAGTCCTT
GAATCACATCTCATTGGTGTGCAGATCAGTTGAGAAATCCCTTGATTTCTACCAGAGTGTTCTTGGGTT
CTTCCCAATTAGGAGTTCTGGCTCCTTTGACTTTAATGGTGCATGGCTATTCAATTATGGCATTGGCATA
CATCTTCTCCAATCTGAAGACCCTGATACCCAAGAAGATCACCCAGATTAACCCCAAGGATAACCAC
