# Supplementary material for: Transcriptomic Analysis Reveals Genes Associated with the Regulation of Peach Fruit Softening and Senescence during Storage
Source: Foods. 2023 Apr 14;12(8):1648. doi: 10.3390/foods12081648 (PMC10137801; doi:10.3390/foods12081648)
Supplement: Supplementary file 1 [file foods-12-01648-s001.zip › Supplementary File 7.pdf]

Supplementary file S7: FPKM and functional description of candidate genes

Gene ID	FPKM of Samples									Functional description
	XC 0 d	XC 3 d	XC 6 d	HY 0 d	HY 3 d	HY 6 d	HJML 0 d	HJML 3 d	HJML 6 d	
Prupe.1G034300	19.15	46.54	60.00	19.80	18.45	19.09	26.90	168.73	201.38	Ethylene response sensor 2-related
Prupe.1G042500	0.62	0.92	0.75	0.40	1.09	1.13	1.37	9.10	12.53	Phosphate transporter PHO1
Prupe.1G412400	0.65	0.76	1.02	1.67	0.75	1.49	3.50	10.52	9.86	Uncharacterized conserved protein
Prupe.1G526700	20.45	30.60	27.53	14.84	17.15	18.74	17.98	96.70	99.85	Ovarian cancer gene-2 protein-related // subfamily not named
Prupe.2G140600	4.34	2.65	3.40	2.41	2.64	3.81	10.27	25.14	11.71	Auxin-regulated protein-related
Prupe.2G176900	0.76	3.98	6.67	0.06	4.97	1.21	126.04	1568.69	2493.94	1-aminocyclopropane-1-carboxylate synthase-like protein 1
Prupe.2G307400	13.27	5.34	2.24	0.66	3.24	0.38	3.47	32.73	16.32	WRKY DNA -binding domain (WRKY)
Prupe.3G024700	0.11	0.29	0.87	0.03	0.03	0.03	0.36	10.73	10.79	Receptor like protein 3-related
Prupe.3G074800	22.97	149.66	171.28	28.51	296.65	151.35	427.27	1105.54	1159.67	Auxin-responsive protein iaa19-related
Prupe.3G098100	2.82	4.43	9.20	0.25	2.85	0.26	2.36	61.42	73.20	WRKY transcription factor 40-related
Prupe.5G054500	97.02	108.09	99.79	115.87	139.53	116.76	146.10	307.77	501.04	Serine/threonine kinase // subfamily not named
Prupe.6G226100	0.08	0.15	0.03	0.01	0.14	0.13	16.28	73.29	70.56	Auxin responsive GH3 gene family (GH3)
Prupe.6G286000	35.62	21.12	12.80	2.94	7.48	2.14	19.33	64.30	58.10	WRKY transcription factor 33 (WRKY33)
Prupe.7G160600	0.51	0.60	1.76	0.21	0.62	0.26	0.94	2.26	9.76	Camp-response element binding Protein-related // subfamily not named
Prupe.7G234800	0.32	3.08	8.40	3.74	6.19	6.36	20.29	160.61	234.97	Auxin-responsive protein IAA15
Prupe.7G244300	8.09	4.66	7.07	11.59	4.32	4.45	5.78	22.34	19.78	EIN3-binding F-box protein (EBF1_2)
Prupe.7G247500	0.07	0.22	0.04	0.02	9.53	0.60	34.31	185.89	62.56	Auxin-responsive protein IAA (IAA)
Prupe.8G153100	0.10	0.49	3.60	0.50	0.41	0.17	0.37	4.47	10.83	Pathogenesis-related protein 1 (PR1)
Prupe.8G153700	0.07	2.61	5.79	0.34	3.00	3.67	0.84	10.81	18.36	Pathogenesis-related protein 1 (PR1)
Prupe.8G153800	129.63	174.69	213.53	16.44	41.88	12.87	183.62	415.70	531.36	Pathogenesis-related protein 1 (PR1)

Note：Function description data was from JGI database（https://phytozome.jgi.doe.gov/pz/portal.html#!info? alias=Org_Ppersica
