# Supplementary material for: Transcriptomic Analysis Reveals Genes Associated with the Regulation of Peach Fruit Softening and Senescence during Storage
Source: Foods. 2023 Apr 14;12(8):1648. doi: 10.3390/foods12081648 (PMC10137801; doi:10.3390/foods12081648)
Supplement: Supplementary file 1 [file foods-12-01648-s001.zip › Supplementary File 8.pdf]

Supplementary file S8 FPKM of 20 candidate genes in figure 7

| Gene name | XC 0 d | | | XC 4 d Control | | | XC 4 d NAA | | |
|---|---|---|---|---|---|---|---|---|---|
| | XCT1 | XCT2 | XCT3 | XCT4 | XCT5 | XCT6 | XCT7 | XCT8 | XCT9 |
| *Prupe.1G034300* | 26.87 | 30.59 | 33.37 | 27.2 | 32.55 | 26.82 | 517.93 | 498.76 | 492.42 |
| *Prupe.1G042500* | 1.47 | 1.72 | 1.72 | 1.01 | 1.13 | 0.74 | 2.6 | 2.85 | 2.64 |
| *Prupe.1G412400* | 0.76 | 0.88 | 0.77 | 1.36 | 1.22 | 1.08 | 8.05 | 8.64 | 9.47 |
| *Prupe.1G526700* | 24.1 | 30.29 | 34.1 | 21.61 | 24.73 | 27.76 | 54.22 | 51.13 | 50.57 |
| *Prupe.2G140600* | 9.14 | 9.96 | 12.6 | 2.98 | 3.8 | 3.19 | 4.42 | 4.83 | 3.32 |
| *PpACS1* | 0.22 | 0.47 | 0.48 | 1.61 | 1.37 | 1.77 | 198.47 | 184.83 | 208.09 |
| *Prupe.2G307400* | 20.89 | 31.77 | 40.87 | 3.88 | 11.63 | 9.88 | 11.66 | 14.44 | 10.57 |
| *Prupe.3G024700* | 0 | 0.04 | 0.04 | 0.13 | 0.31 | 0.19 | 43.29 | 38.42 | 39.35 |
| *Prupe.3G074800* | 16.45 | 18.09 | 18.28 | 131.9<br>1 | 153.44 | 156.87 | 320.08 | 298.68 | 302.57 |
| *Prupe.3G098100* | 1.65 | 4.41 | 6.17 | 5.14 | 15.72 | 12.39 | 75.09 | 73.99 | 68.61 |
| *Prupe.5G054500* | 154.6 | 146.61 | 145.09 | 100.7<br>1 | 110.86 | 116.08 | 282.19 | 276.07 | 275.35 |
| *Prupe.6G226100* | 0.05 | 0.08 | 0.03 | 0.03 | 0.1 | 0.1 | 48.38 | 44.33 | 47.91 |
| *Prupe.6G286000* | 47.92 | 79 | 94.97 | 10.98 | 27.68 | 18.53 | 23.45 | 24.07 | 20.62 |
| *Prupe.7G160600* | 0.57 | 0.58 | 0.36 | 0.23 | 1.28 | 0.81 | 5.22 | 7.9 | 7.5 |
| *Prupe.7G234800* | 0.95 | 1.67 | 1.33 | 5.33 | 9.68 | 6.62 | 292.16 | 266.06 | 256.48 |
| *Prupe.7G244300* | 13.03 | 14.11 | 14.3 | 8.84 | 8.16 | 7.46 | 32.44 | 33.48 | 33.49 |
| *Prupe.7G247500* | 0 | 0.07 | 0 | 0.43 | 1.01 | 0.72 | 35.3 | 34.99 | 31.65 |
| *Prupe.8G153100* | 0.56 | 0.51 | 0.31 | 0.67 | 0.85 | 0.4 | 2.56 | 1.84 | 2.35 |
| *Prupe.8G153700* | 0 | 0.52 | 0.16 | 0.74 | 0.5 | 0.28 | 11.68 | 10.24 | 13.63 |
| *Prupe.8G153800* | 12.31 | 15.52 | 13.02 | 86.55 | 72.7 | 64.28 | 203.99 | 238.48 | 263.31 |

Note: ‘XC’ represents ‘Xia Cui’.
